# Supplementary material for: Adult zebrafish can learn Morris water maze-like tasks in a two-dimensional virtual reality system
Source: Cell Rep Methods. 2024 Sep 23;4(10):100863. doi: 10.1016/j.crmeth.2024.100863 (PMC11573742; doi:10.1016/j.crmeth.2024.100863)
Supplement: Document S1. Figures S1 and S2 and Tables S1 and S2 [file mmc1.pdf]

Cell Reports Methods, Volume *4*

Supplemental information

Adult zebrafish can learn Morris water maze-like tasks in a two-dimensional virtual reality system

Tanvir Islam, Makio Torigoe, Yuki Tanimoto, and Hitoshi Okamoto

Supplemental Information

Supplementary Figures

Figure S1. Persistence and goal headedness in the VMWM task.

Figure S2. Heatmaps of statistical P-values of comparison of persistence at point-places in the VMWM arena.

Supplementary Tables

Table S1. Experimental information and learning performances of the shocked fish in the VMWM.

Table S2. Experimental information and learning performances of the control fish in the VMWM.

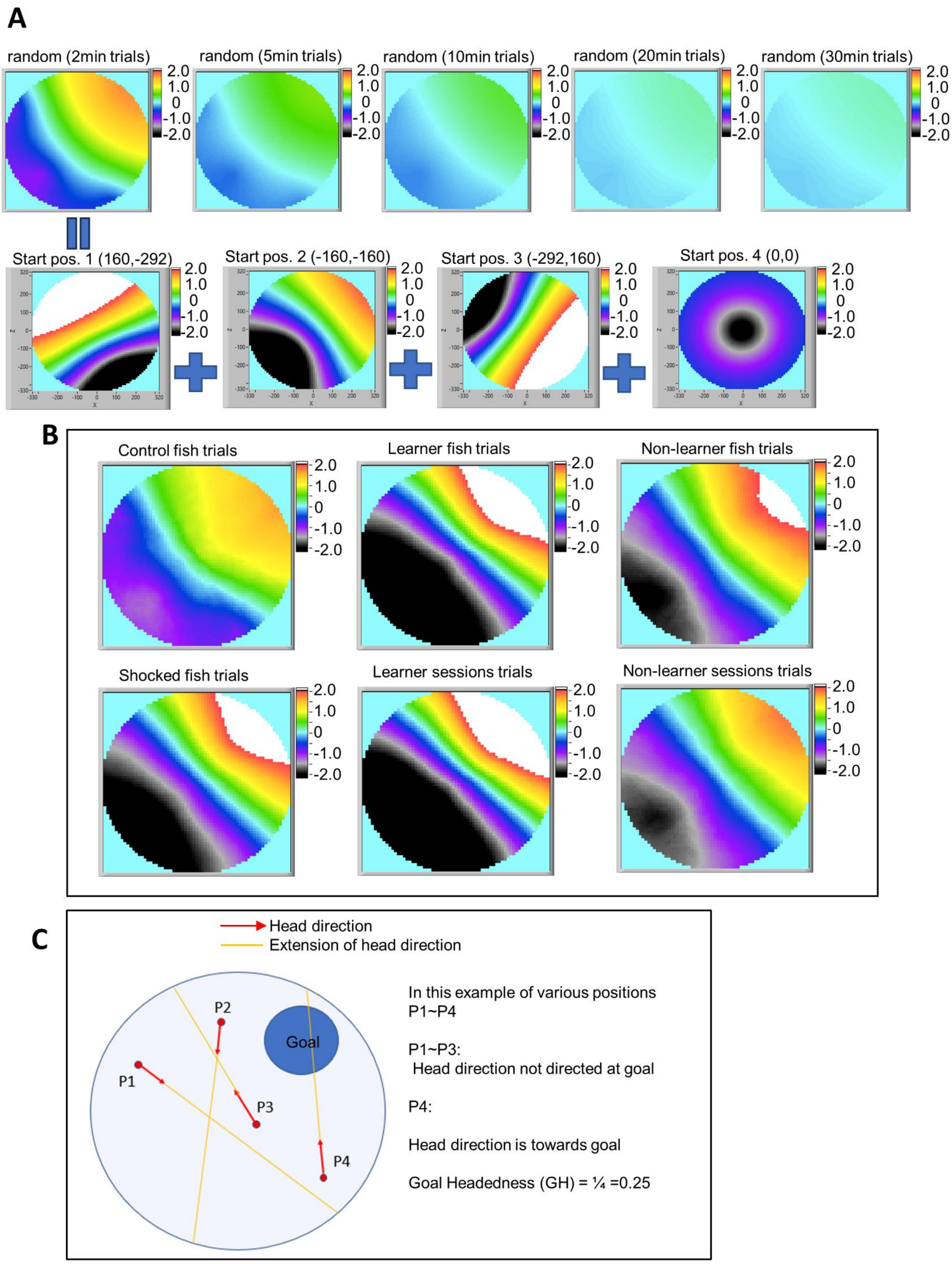
A
random (2min trials)
random (5min trials)
random (10min trials)
random (20min trials)
random (30min trials)
2.0
1.0
0
-1.0
-2.0
Start pos. 1 (160,-292)
Start pos. 2 (-160,-160)
Start pos. 3 (-292,160)
Start pos. 4 (0,0)
B
Control fish trials
Learner fish trials
Non-learner fish trials
Shocked fish trials
Learner sessions trials
Non-learner sessions trials
C
Head direction
Extension of head direction
P1
P2
P3
P4
Goal
In this example of various positions P1~P4
P1~P3:
Head direction not directed at goal
P4:
Head direction is towards goal
Goal Headedness (GH) = ¼ =0.25

Figure S1: Persistence and goal headedness in the VMWM task, related to Figure 5. (A) Persistence maps derived from 1200 simulated VMWM trials, using random movement. The first map from the upper left is for 2-minute trials, which have the same length as trials experienced by fish in VMWM trials. Because of the location of the start positions and the structure of the arena, as shown in the bottom four panels, there is a resultant bias of higher persistence towards the upper right quadrant of the arena. However, as the next four maps show, this bias disappears with increased length of trials. The calculation method of persistence is provided in the equation inside the text box. (B) Persistence heat maps derived by another method [40]. It is notable that the persistence maps look similar in two methods. (C) Explanatory diagram for calculation of goal-headedness (STAR Methods). Among the four positions p1-p4, the head direction during only position p4 is goal-headed, as the hypothetical extension if that head direction goes through the safe zone. In this case of four positions, the goal headedness score is therefore 1/4=0.25.

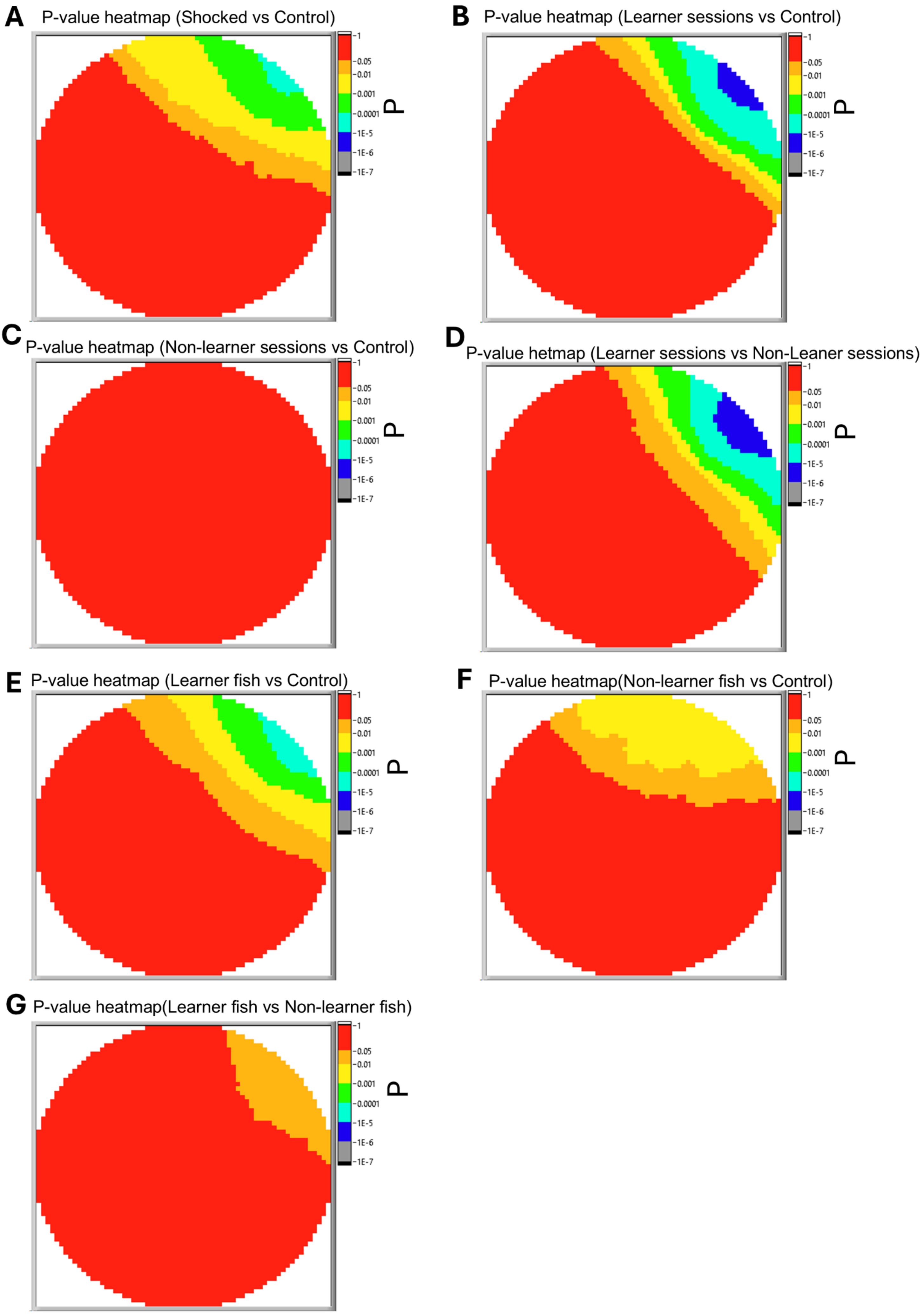
A P-value heatmap (Shocked vs Control)
B P-value heatmap (Learner sessions vs Control)
C P-value heatmap (Non-learner sessions vs Control)
D P-value hetmap (Learner sessions vs Non-Leaner sessions)
E P-value heatmap (Learner fish vs Control)
F P-value heatmap(Non-learner fish vs Control)
G P-value heatmap(Learner fish vs Non-learner fish)
1
0.05
0.01
0.001
0.0001
1E-5
1E-6
1E-7
P

Figure S2: Heatmaps of statistical P-values at the point-places while comparing persistence from different groups, related to Figure 5. (A) P-value heatmap obtained from comparison of persistence in all point-places among the shocked and control fish (lower P-values mean higher significance). Shocked fish showed significantly higher persistence ($p<0.0001$) in the vicinity of the safe zone. (B) Similar P-value heatmap from comparison between learner sessions and control. Rest of the figures also show P-value heatmaps for the following statistical comparisons: (C) Non-learner sessions vs Control. (D) Learner sessions vs Non-learner sessions. (E) Learner fish vs Control. (F) Non-learner fish vs Control. (G) Learner fish vs Non-learner fish

Table S1: Experimental information and learning performances of the shocked fish in the VMWM, related to Figures 2 and 3. The number of trials, number of successes, and success rate are shown for each fish in the shocked group. In each cell corresponding to a fish and a day, a division of the number of success trials by the total number of trials is multiplied by 100 to show the success rate of that fish on that day. A (S) tag (S=Significant) is used if the success rate is significantly higher than control fish, otherwise a notation of (NS) is used. The sessions (day) with a label (S) are considered as “learner sessions” throughout the paper. A shocked fish is assigned an “L” or “learner” tag if the last session(day) of that fish is a “significant” session. Fish with an “L” tag are considered as “learner fish” throughout the paper.

Fish #	Day 1 success rate (SR) %	Day 2 success rate (SR) %	Day 3 success rate (SR)%	Day 4 success rate (SR) %	Learner? Learner=L
01	SR=4/70=5.71<br>P=0.2198 (NS)	SR=12/80=15<br>P=0.060456 (NS)	SR=6/65=9.23<br>P=0.35355 (NS)	N/A	
02	SR=16/70=22.85<br>P=0.020276 (NS)	SR=30/66=45.45<br>2.06E-08(S)	SR=0/60=0<br>P=1.48E-06 (NS)	N/A	
03	SR=19/65=29.23<br>P=0.000613(NS)	SR=31/65=47.69<br>P=4.78E-10 (S)	N/A	N/A	L
04	SR=44/70=62.8<br>2.12E-28(S)	N/A	N/A	N/A	L
05	SR=23/50=46<br>P=2.04E-08 (S)	SR=35/75=46.66<br>P=7.98E-11 (S)	N/A	N/A	L
06	SR=24/60=40<br>P=2.66E-07(S)	SR=32/60=53.33<br>P=5.55E-12(S)	N/A	N/A	L
07	SR=19/60=31.66<br>P=0.000197 (NS)	SR=7/60=11.66<br>P=0.3047(NS)	SR=2/60=3.33<br>P=8.36E-05(NS)	N/A	
08	SR=9/60=15<br>P=0.40721 (NS)	SR=28/60=46.66<br>P=6.03E-09(S)	SR=1/60=1.66<br>P=9.81E-06(NS)	N/A	
09	SR=19/70=27.14<br>P=0.001634 (NS)	SR=15/46=32.6<br>P=0.002164 (NS)	SR=30/51=58.82<br>P=1.23E-08(S)	SR=27/51=52.94<br>P=7.04E-07(S)	L
10	SR=29/54=53.7<br>P=2.26E-12 (S)	SR=17/65=26.15<br>P=0.013616 (NS)	N/A	N/A	
11	SR=23/73=31.5<br>p=4.83E-05(S)	SR=24/55=43.64<br>P=3.46E-07 (S)	SR=16/29=55.17<br>P=6.82E-05 (S)	N/A	L
12	SR=30/50=60<br>P=1.68E-14 (S)	SR=28/50=56<br>P=2.44E-11 (S)	SR=37/53=69.81<br>5.21E-14(S)	N/A	L
13	SR=30/80=37.5<br>P=5.13E-08 (S)	N/A	N/A	N/A	L
14	SR=17/80=21.25<br>P=0.03328 (NS)	N/A	N/A	N/A	
15	SR=34/80=42.5<br>P=1.35E-10 (S)	N/A	N/A	N/A	L
16	SR=48/80=60<br>P=1.90E-40 (S)	N/A	N/A	N/A	L
17	SR=15/80=18.75<br>P=0.10694 (NS)	SR=24/70=34.28<br>P=4.90E-05 (S)	N/A	N/A	L
18	SR=21/80=26.25<br>P=0.001519 (NS)	N/A	N/A	N/A	
19	SR=26/80=32.5<br>P=8.58E-06 (S)	N/A	N/A	N/A	L
20	SR=16/80=20<br>P=0.061609 (NS)	N/A	N/A	N/A	
21	SR=24/65=36.36<br>P=1.49E-06 (S)	SR=28/60=46.67<br>P=6.03E-09 (S)	SR=34/60=56.67<br>P=2.55E-09 (S)	N/A	L
22	SR=17/60=28.33<br>P=0.001695 (NS)	SR=32/60=49.23<br>P=8.84E-11 (S)	SR=25/60=41.66<br>P=0.0003 (NS)	N/A	
23	SR=5/80=6.25<br>P=0.035693 (NS)	SR=1/60=1.66<br>P=0.000675 (NS)	N/A	N/A	
24	SR=19/70=27.14<br>P=0.001634 (NS)	N/A	N/A	N/A	
25	SR=15/62=24.19%<br>P=0.014621 (NS)	SR=4/64=6.25<br>P=0.027971(NS)	SR=45/86=52.32<br>9.39E-11(S)	N/A	L
26	SR=20/70=28.57<br>P=0.000617 (NS)	SR=29/60=48.33<br>P=1.16E-09 (S)	SR=20/60=33.33<br>P=0.020933 (NS)	N/A	
27	SR=6/70=8.57<br>P=0.15905 (NS)	N/A	N/A	N/A	

Table S2. Experimental information and learning performances of the control fish in the VMWM, related to Figures 2 and 3. Number of trials, number of successes, and success rate are shown for each fish in the control group. For any day, the control fish data with highest success rate (fish#01 for day 1 and day 2, fish#04 for day 3) is chosen to compare with any shocked fish data for statistical significance.

Fish #	Day 1 success rate (%)	Day 2 success rate (%)	Day 3 success rate (%)
01	8/60=13.33	9/60=15	3/60=5
02	6/60=10	8/60=13.33	2/60=3.33
03	6/60=10	1/60=1.66	10/60=16.67
04	2/60=3.33	4/60=6.66	13/60=21.67
05	0/80=0	6/70=8.57	N/A
06	2/80=2.5	6/70=8.57	5/70=7.14
07	2/70=2.85	7/70=10	1/70=1.42
08	6/70=8.57	5/70=7.14	4/70=5.71
09	4/70=5.71	4/70=5.71	1/70=1.42
10	5/70=7.14	1/70=1.42	0/70=0
