# Supplementary material for: Adult zebrafish can learn Morris water maze-like tasks in a two-dimensional virtual reality system
Source: Cell Rep Methods. 2024 Sep 23;4(10):100863. doi: 10.1016/j.crmeth.2024.100863 (PMC11573742; doi:10.1016/j.crmeth.2024.100863)
Supplement: Document S2. Article plus supplemental information [file mmc2.pdf]

Cell Reports Methods

Article

Adult zebrafish can learn Morris water maze-like tasks in a two-dimensional virtual reality system

Graphical abstract

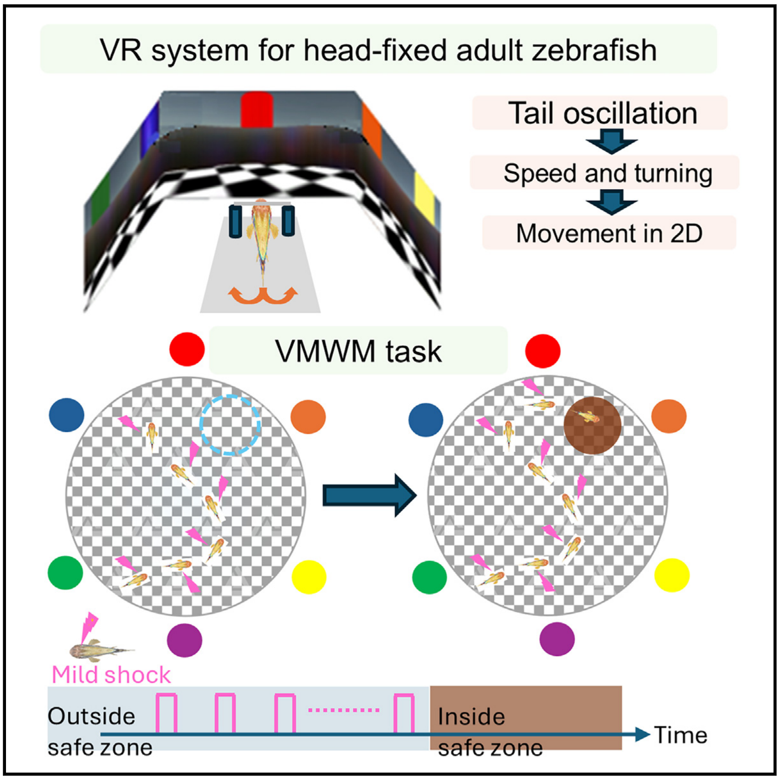

Authors

Tanvir Islam, Makio Torigoe,
Yuki Tanimoto, Hitoshi Okamoto

Correspondence

tanvir.islam@riken.jp (T.I.),
hitoshi.okamoto@riken.jp (H.O.)

In brief

In this article, Islam et al. design a Morris water maze-like task to investigate the spatial learning ability of zebrafish using a virtual reality system for adult zebrafish. This system enables optical imaging investigations of neural mechanisms for space cognition.

Highlights

- A virtual Morris water maze (VMWM) task is established for adult zebrafish
- Zebrafish show increased spatial learning performance over days
- Path straightness and goal headedness are enhanced in VMWM task

Islam et al., 2024, Cell Reports Methods *4*, 100863
October 21, 2024 © 2024 The Author(s). Published by Elsevier Inc.
https://doi.org/10.1016/j.crmeth.2024.100863

CellPress

Cell Reports Methods

CellPress
OPEN ACCESS

Article

Adult zebrafish can learn Morris water maze-like tasks in a two-dimensional virtual reality system

Tanvir Islam,[1,*] Makio Torigoe,[1] Yuki Tanimoto,[1,2] and Hitoshi Okamoto[1,2,3,4,*]
[1]RIKEN Center for Brain Science, 2-1 Hirosawa, Wako-shi, Saitama 351-0198, Japan
[2]School of Advanced Science and Engineering, Waseda University, 2-2 Wakamatu-cho, Shinjuku-ku, Tokyo 169-8555, Japan
[3]Institute of Neuropsychiatry, 91 Benten-cho, Shinjuku-ku, Tokyo 162-0851, Japan
[4]Lead contact
*Correspondence: tanvir.islam@riken.jp (T.I.), hitoshi.okamoto@riken.jp (H.O.)
https://doi.org/10.1016/j.crmeth.2024.100863

MOTIVATION Large-scale calcium imaging has become a prominent method in neuroscience, providing insight into brain functions during navigation, social interaction, decision-making, and complex innate behavior. This method remains underutilized in adult zebrafish, for which a combination of virtual reality (VR) systems and behavioral experiments in a head-fixed state is necessary. Furthermore, to investigate the properties of neurons during complex tasks, including navigation and social interaction, it is necessary to build VR systems that allow effective movement in a two-dimensional plane. To address this, we have created a two-dimensional VR system for adult zebrafish. To validate the system and investigate the spatial learning ability of zebrafish, we designed a virtual Morris water maze-like experiment in which zebrafish had to forage through a circular VR arena to find an invisible safe zone while receiving a periodic mild electric shock. We thus demonstrate a method for the investigation of the neural coding of space in zebrafish brain that will enable the future incorporation of advanced optical imaging.

SUMMARY

Virtual reality (VR) has emerged as a powerful tool for investigating neural mechanisms of decision-making, spatial cognition, and navigation. In many head-fixed VRs for rodents, animals locomote on spherical treadmills that provide rotation information in two axes to calculate two-dimensional (2D) movement. On the other hand, zebrafish in a submerged head-fixed VR can move their tail to enable movement in 2D VR environment. This motivated us to create a VR system for adult zebrafish to enable 2D movement consisting of forward translation and rotations calculated from tail movement. Besides presenting the VR system, we show that zebrafish can learn a virtual Morris water maze-like (VMWM) task in which finding an invisible safe zone was necessary for the zebrafish to avoid an aversive periodic mild electric shock. Results show high potential for our VR system to be combined with optical imaging for future studies to investigate spatial learning and navigation.

INTRODUCTION

Virtual reality (VR) is a suitable tool to investigate the neural coding of interactions with the environment while allowing for quantitative measurements of cognitive behavior in well-controlled tasks, including decision-making, spatial cognition, learning and memory, and social interaction, and has been utilized across species.[1–10] Head fixation in VR enables the recording of activities from hundreds of neurons in wide-scale calcium imaging that cannot be achieved in freely moving animals.[2,11–15] Among several species, VR has been mainly used with rodents, for example, for spatial navigation tasks in head-fixed mice that allow the use of intracellular recording and two-photon microscopy.[3,16–22] However, while many of the linear virtual tracks with a single dimension[3,16,18–20,22–24] or body-fixed rats[25,26] could reveal neuronal properties like place or grid cells, the two-dimensional (2D) firing patterns of cells observed in real-world open arenas were missing. Other systems using a body-suit or "jacket"[27] to fixate the rodent body were unsuitable for optical imaging due to head movement. A method using a bearing device to allow head rotation[27] was proposed to be compatible with calcium imaging. However, an optical imaging study based on such a system is yet to be reported.

Small teleosts, like zebrafish, are also suitable for optical imaging along with a behavior paradigm in VR,[14,28–33] although mainly larval-stage fish have been used so far due to the technical

© 2024 The Author(s). Published by Elsevier Inc.
This is an open access article under the CC BY license (http://creativecommons.org/licenses/by/4.0/).

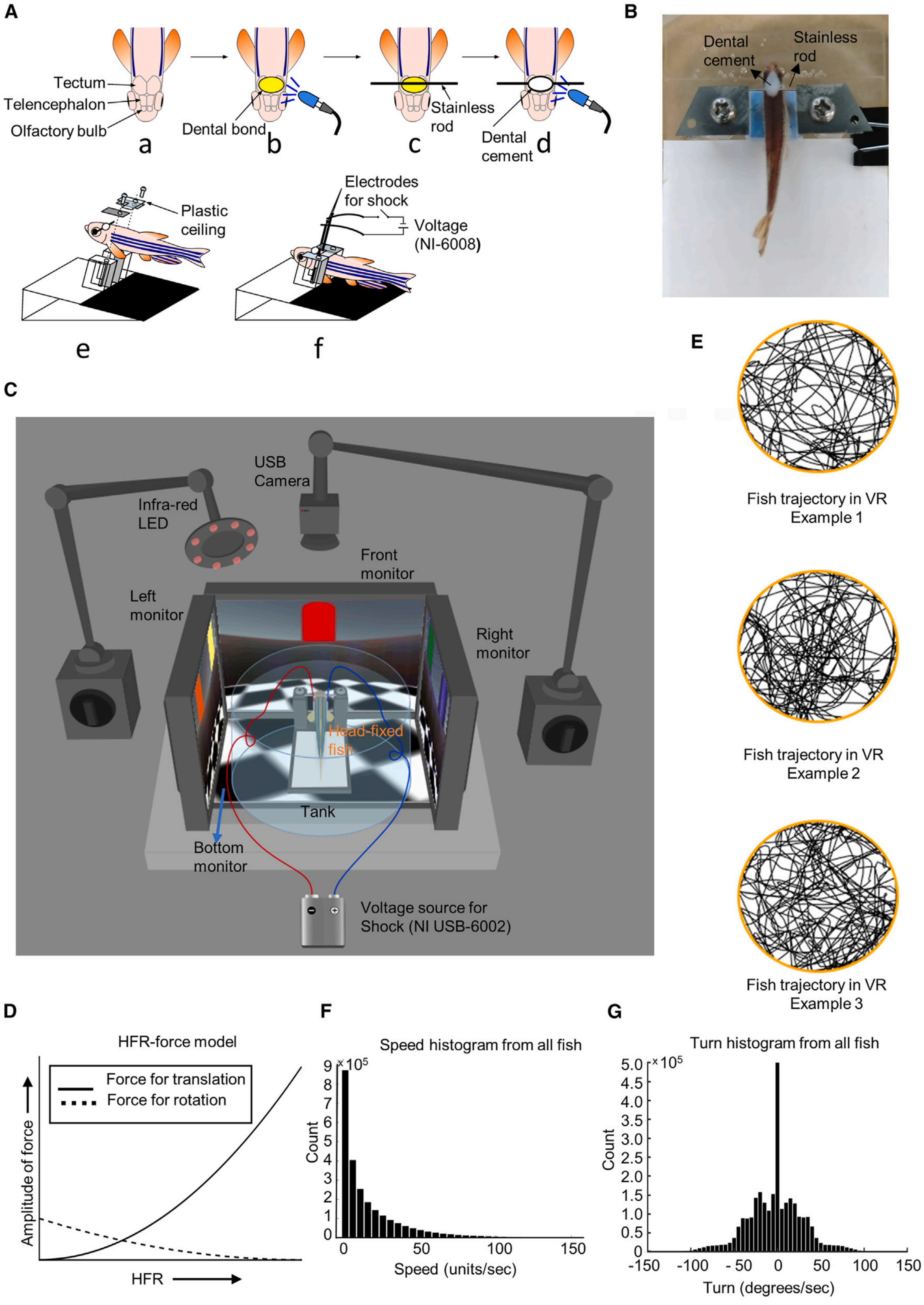

(legend on next page)

advantage of recording from the whole brain. In contrast to the often simplistic and reflexive behavioral patterns exhibited by larvae, adult zebrafish have a broader behavioral range that includes social interactions, complex innate behaviors, and higher learning ability in cognitive tasks.[31,34] Adult zebrafish brains are still sufficiently small to perform high-resolution optical imaging, thus making them a promising animal model for investigating higher brain functions. To investigate the neural correlates of decision-making in a task-specific behavioral paradigm, we have designed a GO-NOGO task in a VR system with a linear track[1] for head-fixed zebrafish, with which neural ensembles in telencephalon that encode task rules attached to specific colors and scenery flow prediction error (SFPE) could be identified. However, more complex tasks involving spatial and cue learning and social interaction require 2D VR for zebrafish, which allows forward movement and turning to forage through 2D space. A 2D VR system for head-fixed adult zebrafish was proposed by Huang et al.[35] where free movement by adult zebrafish in a naturalistic VR was used to study differential and directional behavior responses to visual stimuli as well as the effect of unexpected sensory input that evoked neuronal responses in multiple brain regions to encode the mismatch between the expected and actual sensory inputs, i.e., prediction error. Keeping in mind the necessity of a VR system for 2D movement in virtual space, we created a VR system to allow movements in two dimensions with real-time calculations for forward speed and turning. The mechanism of our system uses a different set of real-time calculations from the previously proposed methods, i.e., rather than calculating the amount of displacement and rotation in two dimensions, our system calculates the instantaneous forces for forward translation and rotation that are applied on an object corresponding to the first-person view in VR. This mechanism allows the use of sophisticated physics engines of game engines like Unity, which provides the natural inertia effects that the fish experiences in its movements in VR.

To check the spatial learning ability of zebrafish in VR, we designed a virtual Morris water maze-like (VMWM) task, which is a mimicry of the MWM test[36] originally developed for rodents. In this task, we made zebrafish-specific modifications like introducing a periodic mild electric shock to create an aversive condition from the beginning of the trial and eliminating the shock after the zebrafish reached a safe zone, which was invisible at the beginning. Thus, avoidance of the periodic shock acted as a reward for zebrafish to search for the invisible safe zone. Here, we present extensive analysis of the behavior of a group of zebrafish that performed the VMWM task. The results described below show the ability of zebrafish to adapt to our VR system and show considerable spatial learning performance.

RESULTS

A 2D VR system for head-fixed adult zebrafish

We created a 2D VR system on the basis of our previously proposed linear VR system[1] by partial modification of hardware but new software implementations (STAR Methods). The zebrafish was head fixed with a metallic rod of stainless steel with a 0.5 mm circumference (Figures 1A and 1B) instead of the metallic bar used previously[1] to increase tail movement ability. Four monitors surrounded the head-fixed zebrafish placed inside a circular-shaped transparent tank filled with water to create the VR environment (Figure 1C). Fish tail movement was continuously recorded with a web camera placed above the fish. A fast Fourier transformation (FFT) of tail oscillation was conducted to extract the high-frequency ratio (HFR) in every time step (denoted in Equations 2 and 3 in the STAR Methods as *HFR(t)*), which was used to calculate the forces for forward movement ($Force_{translation}$) and turning ($Force_{rotation}$). These two forces for forward speed and turning changed non-linearly with changing HFRs (Figure 1D) because of imposed non-linearity with the power of two, which is used to strengthen the effect of mutual exclusiveness between the two forces. A higher HFR contributed to a higher force for speed and a lower force for turning, and vice versa. We tested if zebrafish could adapt to the mechanism of VR and learn to move around the 2D VR space without constraints by letting head-fixed zebrafish free swim in a circular arena. Example trajectories of such 1 h of free swimming are depicted in Figure 1E, in which zebrafish show movements evenly in all parts of the arena. The distribution of speed and turning angle from movement data pulled from all zebrafish used in this paper is shown in Figures 1F and 1G. The shape of the speed histogram (Figure 1F) is comparable to the single zebrafish speed distribution in the real world shown previously.[37] However, in our VR environment, the peak of the histogram shifted left to a speed of zero, probably due to head fixation and a lack of real-world cues. The shape of the turning angle distribution (Figure 1G) is comparable to the real-world turning histogram shown previously[38] in the presence of projected light, which may have created a similar environment to light emitted from the four VR displays in our experiments.

Figure 1. Fixation procedure of zebrafish in VR system, mechanics of movement, and behavioral histograms of zebrafish

(A) Fixation procedure of zebrafish in virtual reality (VR) setup. A metallic rod is attached to the skull with dental cement. The two ends of the rod are fixed on two transparent plastic ceilings on both sides of the fish with screws.

(B) Zebrafish head fixed with a metallic rod over the head. The two ends of the rod are attached with thin structures placed on a white platform, which is used to extract the fish body from the image.

(C) Schematic of the VR experiment setup. A single adult zebrafish, head fixed on a platform with a white background, is placed inside a circular transparent tank filled with water. VR is generated by displaying scenes on four left, front, right, and bottom displays. The fish body is illuminated with an infrared light-emitting diode (LED), and tail movement is detected by a camera with a band-pass filter corresponding to the frequency of the LED.

(D) Non-linear curves of force values for speed and turn with varying HFRs. The force for speed increases with increasing HFRs, while the force for turn decreases.

(E) Example trajectories of free swimming by three zebrafish in a circular virtual arena of 335 units of radius. Swimming time is 1 h in each case.

(F) Speed histogram derived from all fish used in the two experiments in this paper. The unit of speed is noted as units/sec, with "units" corresponding to units of the cartesian axis system in Unity.

(G) Turn histogram derived from all fish used in the two experiments in this paper. Positive and negative values of turn correspond to clockwise and anti-clockwise turns, respectively.

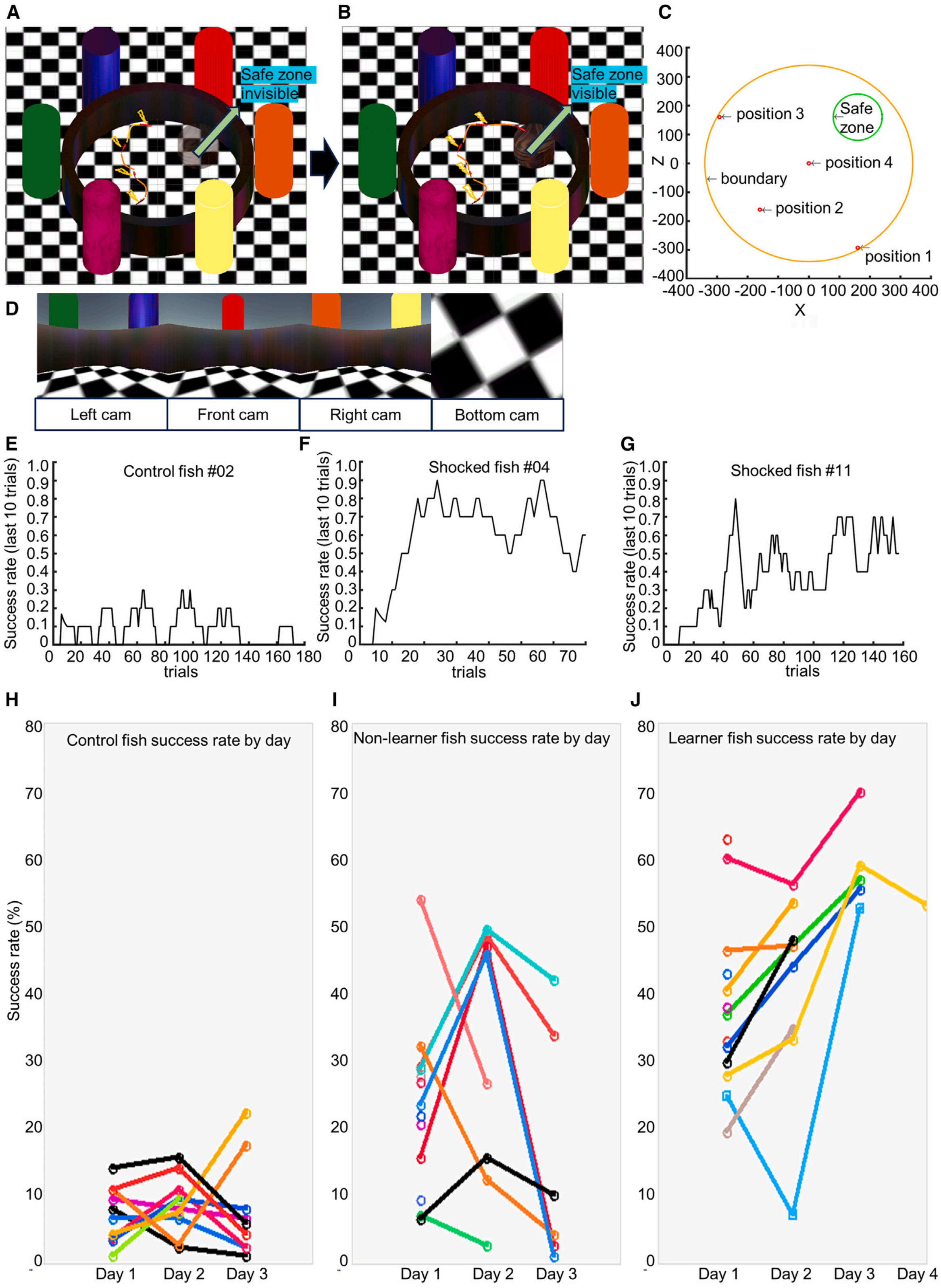

(legend on next page)

The symmetric shape of the turning histogram implies an equal frequency of the left and right bends of the tail across the population. However, a bias toward either could be observed in individuals throughout the experiment time.

A VMWM task can be learned by zebrafish

To validate our VR system and investigate if zebrafish can exploit a spatial memory, we created a VMWM (STAR Methods) task by mimicking the original MWM.[36] In every trial of this task, zebrafish started from one of the four start positions (Figure 2C) in a circular virtual arena (Figures 2A–2D) and navigated to find an invisible safe zone using six visual cues placed outside the arena as references. One trial was of 2 min in length, followed by a 30 s inter-trial interval. To create an aversive condition within the water, which is the natural habitat of fish, we gave the fish a mild (0.5–1 V) periodic electric shock starting from the onset of trials. Zebrafish kept receiving the electric shock until they could reach an invisible safe zone, in which case the shock was eliminated. Therefore, avoidance of this periodic shock was the reward for the zebrafish in this experiment. If the zebrafish reached the safe zone and stayed there for five consecutive seconds, the trial was considered a success. A total of 10 control fish that did not receive the periodic electric shock and thus did not face any aversive condition were used in the study, along with 27 shocked fish that received an electric shock. For each day's training, the number of success trials was divided by the number of trials to find the success rate of all control (see Table S2) and shocked fish (Table S1). A total of 28 sessions out of 56 sessions from 27 shocked zebrafish were found to be "learner sessions" and 14 out of 27 shocked zebrafish were assigned a "learner" (L) label (see STAR Methods).

The temporal aspect of learning performances of zebrafish from the shocked group was expressed by the learning curves, in which each data point shows the success rate of last ten trials within a range of 0–1. Examples of learning curves from one control fish (3 days, Figure 2E) and two shocked fish (1 day, Figure 2F, and 3 days, Figure 2G, respectively) are shown. Figure 3A shows the average success rate of control and shocked fish from days 1–3. The success rate of shocked fish was significantly higher than control fish on days 1 and 2 (t test, p [day 1] = 0.0000180302, T-stat = -4.96132, p [day 2] = 0.0000395339, T-stat = -4.97722). However, due to some fish not moving much on day 3, significance was not reached (p [day 3] = 0.00382971, T-stat = -3.29248). The day-to-day average success rate of learner fish (Figure 3B) and learner sessions (Figure 3C) increased over the days, though statistical significance ($p < 0.0001$) was not observed. Moreover, the success rates for all four starting positions, which were determined by pooling trials with the same starting position and same day from all shocked fish and calculating the day-wise and place-wise success rates, showed a day-to-day increase in both learner fish (Figure 3D) and learner sessions (Figure 3E), though no statistical test was conducted among these single success rate values. A comparison of success rates for the four starting positions did not reveal any bias toward one specific start position in either learner fish (Figure 3F) or learner sessions (Figure 3G).

To check whether there is any difference between the control and shocked groups, we gathered frame-wise speed data from all trials of all control fish and all trials of all shocked fish. Based on these data, we could generate probability distributions of speed for the two groups (Figure 3H). In comparison of the probability distributions, shocked fish in the VMWM showed significantly higher speed than control fish (Kolmogorov-Smirnov test, $p = \sim 0$). To check if fish learned to be in the safe zone for at least 5 s, we calculated the average speed values from 3 s before the entry to the safe zone to 5 s after the entry in all success and failed trials (Figure 3I). Deacceleration after entering the safe zone was observed in both cases, with higher average speeds in failed trials.

Trajectories of VMWM show an increase of both average SI and higher SI percentages for all four start positions

To investigate how zebrafish got used to the VR system and displayed spatial learning, we looked at the VMWM trials of shocked fish that resulted in success. The trajectories of all success trials, sorted by start position and day, from all shocked fish are shown in Figure 4A. Adaptation to the movement mechanism and spatial learning ability to locate the platform were manifested in the average straightness index (SI; Equation 4 in the STAR Methods) of all shocked fish (Figure 4C), which showed a significant increase of the SI on days 2 and 3 (p [days 1–2] = 1E$-$09, Z-stat = -5.99763, p [days 1–3] = 3.8E$-$08, Z-stat = -5.37647). Moreover, the percentage of high SI (SI > 0.5) also increased day by day in shocked fish (Figure 4D). Interestingly, the average SI increased over days for all four start positions (Figure 4B, top row), with significant increases over days in some cases (p [days1–2] = 7.07E$-$05, Z-stat = -3.80566 for position 1; p [days 1–3] = 4.95E$-$06, Z-stat = -4.41943 for

Figure 2. VR arena for VMWM task and example learning curves

(A) VMWM arena with black boundary wall and the safe zone (brown platform) inside. Six pillars of different colors surround the arena as a distal cue outside the arena. The zebrafish's position is shown with a red shape. The orange line shows the trajectory of movement. The fish receive a periodic shock (shown in yellow) until it reaches the safe zone, which is not visible until the fish reaches it.

(B) Continuation of (A). As the fish reaches the safe zone, the safe zone becomes visible, and the fish does not receive a periodic shock if it is inside the safe zone.

(C) Size and start positions inside the VMWM arena. The start position of a trial is randomly decided.

(D) VR scene of the VMWM arena, which is divided into four equal sizes and displayed on four displays (left, front, right, and bottom display).

(E) Moving success rate (success rate of last ten trials) of a control fish (fish #2) over three days.

(F) Moving success rate of a shocked fish (fish #04) in a single day.

(G) Moving success rate of a shocked fish (fish #11) over 3 days.

(H) Success rates of all control fish over days. Circles denote the success rates, and lines connect the same fish over days. Different colors are used to show different fish in the group.

(I) Success rates of thirteen non-learner fish.

(J) Success rates of fourteen learner fish.

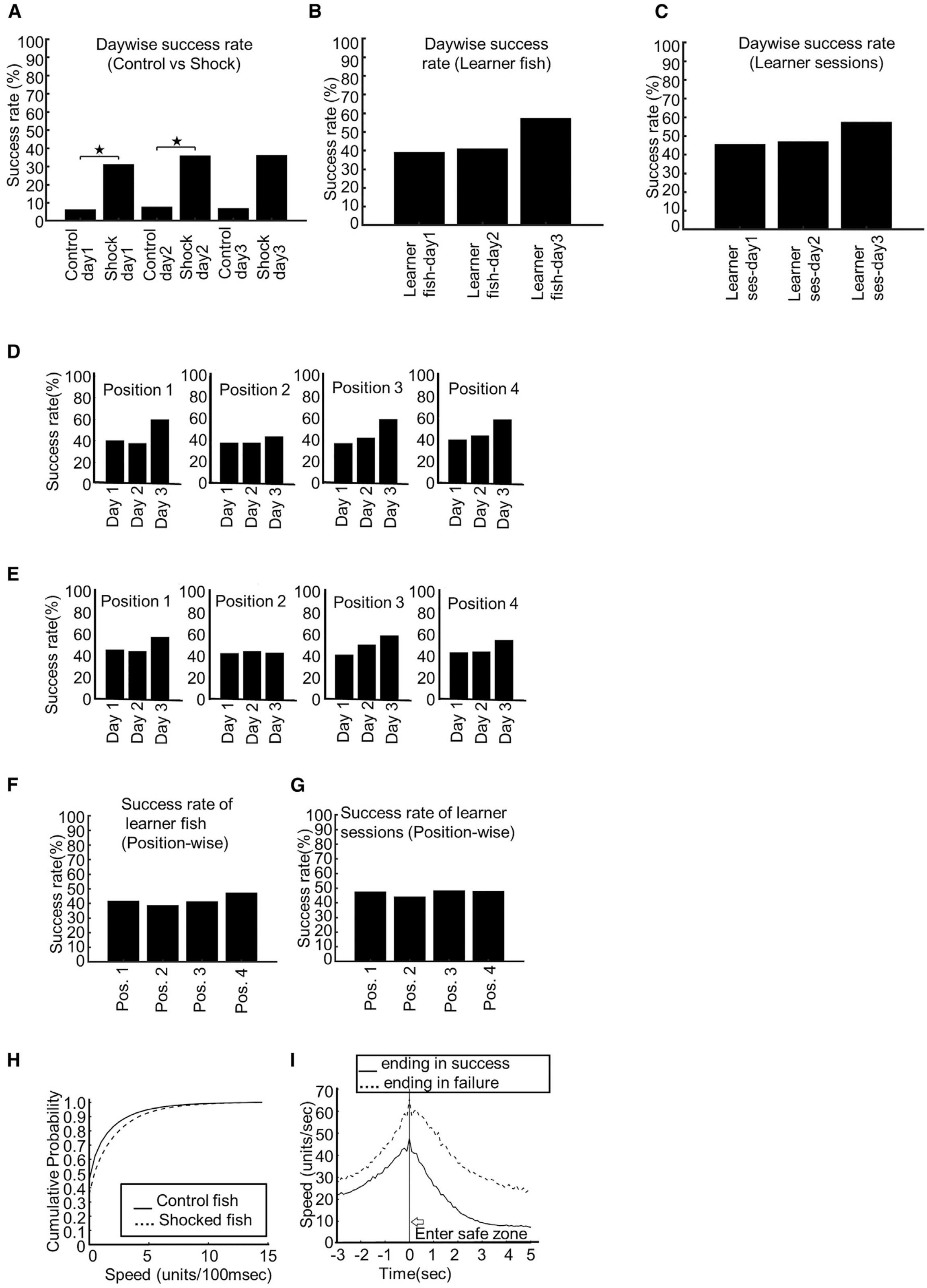

(legend on next page)

position 3; p [days 1–3] = 8.25E−05, Z-stat = −3.76725 for position 4). Furthermore, the percentage of high-SI success trials also increased for all start positions (Figure 4B, bottom row) over days.

Time and travel distance for success trials decrease in learner fish over days

We calculated the time the zebrafish required in each success trial to meet the success criteria. Time required for success decreased significantly on the third day (Figure 5A, top) in learner fish (p [days 1–3] = 1.4118E−07, Z-stat = 5.134857; p [days 2–3] = 2.50277E−05, Z-stat = 4.055368). However, no such decrease was observed in non-learner fish (Figure 5A, bottom). A day-wise reduction of cumulative distance (STAR Methods) from the safe zone center was observed in learner fish (Figure 5B, top), with a significant decrease on the third day (p [days 1–3] = 0, Z-stat = 7.164301; p [days 2–3] = 4.433E−09, Z-stat = 5.751109). Again, such a decrease was not observed in non-learner fish (Figure 5B, bottom). The day-to-day success conversion rates (STAR Methods) of non-learner fish (Figure 5C, bottom) and learner fish (Figure 5C, top) are calculated by gathering day-wise trials from the learner and non-learner fish. These are single numbers and not applicable for statistical significance measurements. A day-to-day increase of the successful conversion rates was observed in the learner fish.

Persistence toward vicinity of the safe zone is visually quantified in VMWM learning

Persistence maps (Equation 5 in the STAR Methods) calculated from VMWM control fish trials and shocked fish trials are shown in Figure 5D. While the persistence map from control fish (Figures 5Da) does not show any significant increase of persistence in the vicinity of the safe zone compared to the map from random trials (Figure S1A, top row, leftmost map), significantly higher persistence values in the vicinity of the safe zone can be seen in the map from shocked fish (Figure 5Dd). Moreover, persistence maps from learner fish trials (Figure 5Db) and learner session (Figure 5De) trials show even higher persistence near the safe zone, and even non-learner fish trials (Figure 5Dc), as well as non-learner session trials (Figure 5Df), show higher persistence values compared to the control fish persistence map. To check if the persistence in the point places vary significantly over the control and shocked groups of fish, we compared the persistence values at every point place from fish of the control and shocked groups (Wilcoxon rank-sum test) and obtained p value maps (Figure S2) that show the presence of significantly higher persistence at the vicinity of the safe zone in the case of the shocked fish (Figure S2A) compared to the control fish. Similar statistical significance can be observed when the learner fish (Figure S2E) and learner session (Figure S2B) data are compared against the control fish data.

We also used a different method to calculate persistence,[39] in which persistence is defined with the following Equation 1:

$$P = \frac{\frac{G}{N} - R}{1 - R} \qquad \text{(Equation 1)}$$

where P is persistence, G is the number of moves toward the place, N is the number of total moves, and R is the probability of a random move being a move toward the place. This method is based on the fraction of moves toward the specific place rather than the net sum of distance toward the place (Equation 5). However, using this method (with $R = 0.5$), similar persistence maps are found, as shown in Figure S1B. Thus, persistence maps highlight zebrafish's higher motivation and effort from the shocked group to reach the safe zone.

GH is strongly observed in VMWM learning

To investigate how much zebrafish tended to head toward the safe zone, we calculated the goal headedness (*GH*) values. Besides persistence, *GH* is another metric that shows to what extent zebrafish are headed toward the safe zone. In Figure 5E, the mean *GHstatic* values derived from shocked fish trials were significantly higher (t test, p = 6.87229E−36, T-stat = −12.5958) than both control fish trials and random simulations, in which 1,200 trials were simulated. Furthermore, the mean *GHstatic* values from learner fish trials and learner session trials were also significantly higher than control fish (control vs. learner fish: p = 5.34141E−52, T-stat = −15.4088; control vs. learner sessions: p = 1.44144E−57, T-stat = −16.3453). Similar results were observed in the *GHmove* (Figure 5F) values, in which shocked fish had a significantly higher value (p = 1.84805E−58, T-stat = 16.3113) than control fish. Besides, learner fish and learner sessions also had significantly higher *GHmove* values (control vs. learner fish: p = 3.49773E−85, T-stat = −20.067; control vs. learner sessions: p = 6.49783E−114, T-stat = −23.4674).

Figures 5G and 5H show the cumulative probability distribution of *GHstatic* and *GHmove* values. A Kolmogorov-Smirnov test shows that in the case of both distributions of *GHstatic* and *GHmove*, higher values are obtained in VMWM-shocked trials compared to VMWM-control trials (Kolmogorov-Smirnov test, p = 1.72559E−46 for *GHstatic*; p = 1.31816E−39 for

Figure 3. Comparison of success rate between groups and over days and start positions

(A) Comparison of day-wise success rates of the control and shocked groups in VMWM task. A statistically significant increase in success rate is observed on days 1–2.

(B) The day-wise average success rate of learner fish from the shocked group shows increase in performance over days.

(C) The day-wise average success rate of learner sessions from shocked fish shows increases in performance over days.

(D) The day-wise success rate of trials pooled from all learner fish for four start positions.

(E) The day-wise success rate of trials pooled from all learner sessions for four start positions.

(F) The success rate of trials pooled from all learner fish for four start positions.

(G) The success rate of trials pooled from all learner sessions for four start positions.

(H) The cumulative probability distribution of speed of the control and shocked groups in VMWM task.

(I) Speed of zebrafish in VMWM task, from 3 s before entering the safe zone to 5 s after (total 8 s). The onset of being in the safe zone is denoted by a vertical line and arrow. Two different lines (solid and broken) show the averaged data of all success trials and all failure trials, respectively.

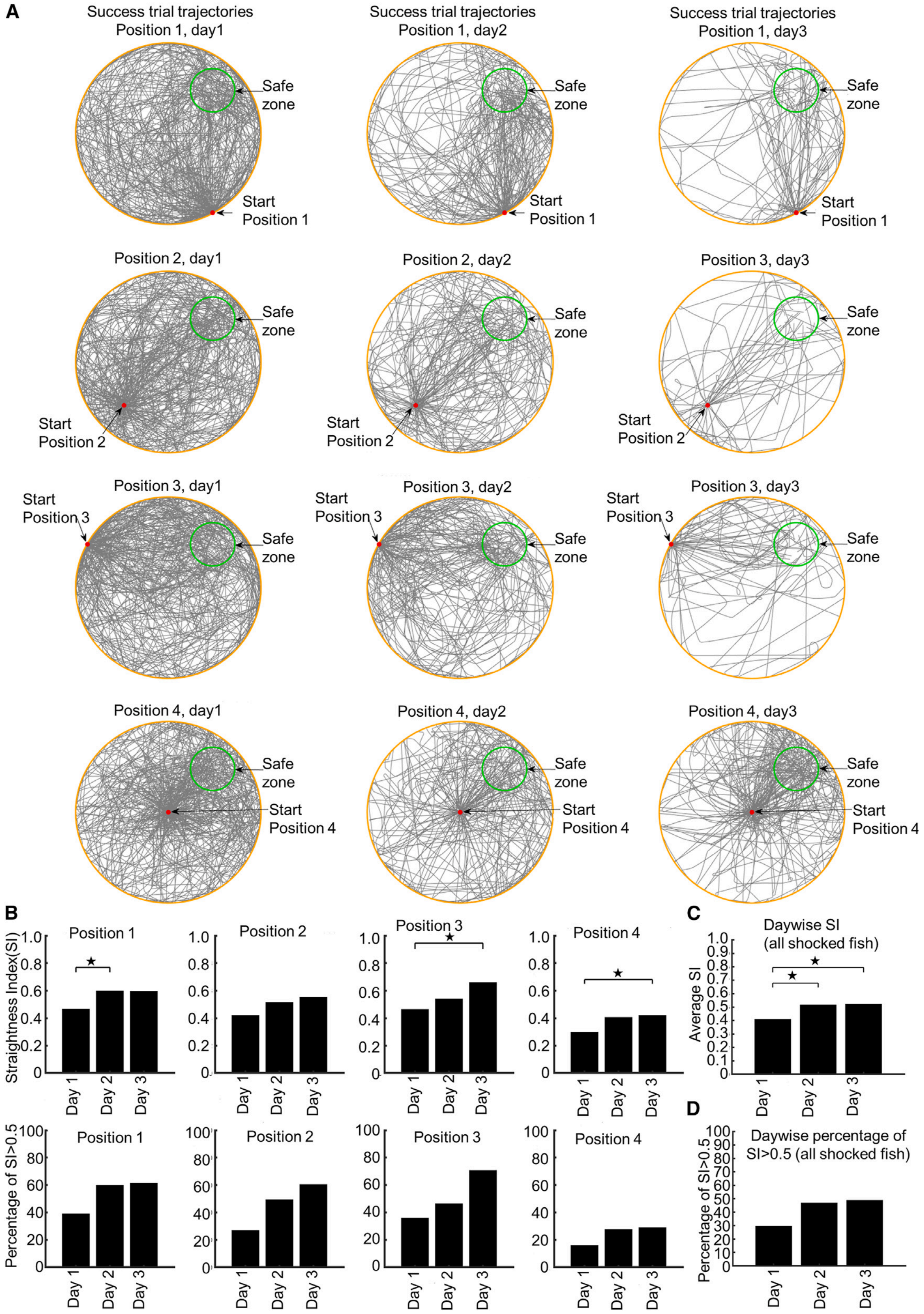

(legend on next page)

GHmove). Similarly, cumulative probability distributions of *GHstatic* and *GHmove* from both learner fish and learner sessions incorporated significantly higher values than control fish (*GHstatic*: control fish vs. learner fish: $p = 3.80538E{-}74$ and control fish vs. learner sessions: $p = 4.73704E{-}81$; *GHmove*: control fish vs. learner fish: $p = 3.11037E{-}71$ and control fish vs. learner sessions: $p = 9.3832E{-}91$). Apart from persistence, the *GH* metrics thus provide intriguing insight regarding goal-directed behavior in the VMWM task.

DISCUSSION

Following our previous work,[1] we created a 2D VR system to allow movement in two dimensions with an aim to enable many future experiments that, together with two-photon imaging, can provide a valuable understanding of the neural coding of spatial learning, navigation, decision-making, social interaction, etc., in adult zebrafish. Real-time analysis of tail movement oscillation was used in our model with other parameters to determine force components for forward translation and rotation, which were then applied to objects in VR using the physics of Unity's game engine The mutual exclusiveness of these two force components ensured that zebrafish could swim straight (high HFR) with a negligible change in direction and turn (low HFR) with negligible translation in the turning direction. In our VR, calculating these two forces and exploiting Unity's physics engine to create the natural inertia effect in speed and turning are distinct differences compared to a previously published 2D VR system for zebrafish,[35] in which forward translation was calculated by adding a forward translation amount each time the tail curvature crossed a midpoint (zero crossing), and the amount of rotation was calculated by the curvature of the tail. Though a direct comparison between the two VR systems is beyond the scope of this paper, we think zebrafish can swim straighter in our VR system using a higher HFR, whereas it may be difficult to swim straight in the mechanism described in the previous paper[35] when there are larger and stronger tail oscillations with higher amplitude, possibly causing some number of alternating rotations in both directions. Due to the mutual exclusiveness of forward translation and rotation in our system (Figure 1D), only negligible rotations can happen in the above-mentioned scenario. Similarly, zebrafish can turn considerably (low HFR) while having only a negligible amount of forward translation in our system. As we update the midpoint continuously, both the forward translation and rotation can be calculated, even in the time periods in which zebrafish bend their tail in one direction and do not cross the midpoint while still oscillating within that side. Besides the normal symmetrical tail oscillation, this one-sided asymmetrical oscillation can be observed occasionally in head-fixed zebrafish in our system. The free-swimming data (Figure 1E) and the subsequent VMWM performance show that zebrafish can adapt to our proposed VR system by moving around the arena evenly, which manifests as zebrafish adapting to our VR system by learning the mechanism of movement.

Besides, the use of the Unity game engine makes it easier to create highly sophisticated and realistic VR scenarios using commercially available or custom 3D models.

As shown in our previous work[1], the head-fixation method we used generates minimal head motion. With extensive imaging, we previously observed that the remaining effects of head motion could be corrected with standard image registration, which makes our system suitable for optical imaging instruments like a two-photon microscope. However, applying the two-photon microscope to acquire images from deeper parts of telencephalon as well as regions like optic tectum, striatum, basal ganglia, etc., poses a strong challenge, and we certainly think that with long-wavelength three-photon microscopy,[40] it will be possible to measure calcium transients deep into these brain regions with relative ease.

We performed the VMWM experiment to investigate spatial learning and navigation ability. In this experiment, we introduced a periodic mild electric shock as an aversive environmental condition for the head-fixed zebrafish that could get rid of the shock by finding and reaching a safe zone that could be located with reference to distal visual cues. Whenever the zebrafish reached the safe zone, the electric shock was stopped, thus making it a zebrafish equivalent of rodents reaching a hidden platform to get rid of the aversive environmental condition, like being in water. Thus, the VMWM task is a zebrafish-specific mimicry of the original MWM task while keeping the essential protocols[41] with the same essence. A MWM-like spatial navigation task was learned by mice in VR,[42] though some aspects, like the initial visibility of the goal and the absence of a requirement to spend a few seconds inside the goal, show some deviation from the original MWM. In our VMWM task, the safe zone was initially invisible at the start of each trial and became visible only when the fish reached the safe zone and remained in it, therefore providing a visual confirmation to the zebrafish. We introduced this feature as an alternative to the somatosensory confirmation of the goal platform's position that rodents receive when they reach and get on the platform in real-world experiments.

In the MWM,[41] learning the location of the safe zone is considered place or spatial learning, and probe or transfer trials (with the platform absent) are administered afterward to check the reference memory at the end of learning. On the other hand, attributes of associative learning can be mixed with spatial learning while providing visual or somatosensory confirmation of the safe zone, and in this case, the probe tests can reveal the extent of spatial learning achieved. Contrary to the real-world MWM, where rodents can learn the spatial location of the safe platform

Figure 4. Trajectories of success trials in VMWM and straightness of path

(A) Superimposed trajectories (in black lines) of the success trials pooled from all fish from the shocked group. Each row corresponds to trajectories of the success trials with the same start position over days 1–3. The safe zone is shown as a green circle.

(B) Top row: the day-wise average SI of success trials pooled from all fish of the shocked group for each start position. Significant differences over days can be observed. Bottom row: percentage of high SI (SI $\geqq$ 0.5) in the day-wise success trials pooled from all shocked fish for each start position.

(C) The day-wise average SI of success trials from all fish from shocked group.

(D) The day-wise percentage of high SI (SI $\geqq$ 0.5) in success trials from all fish from shocked group.

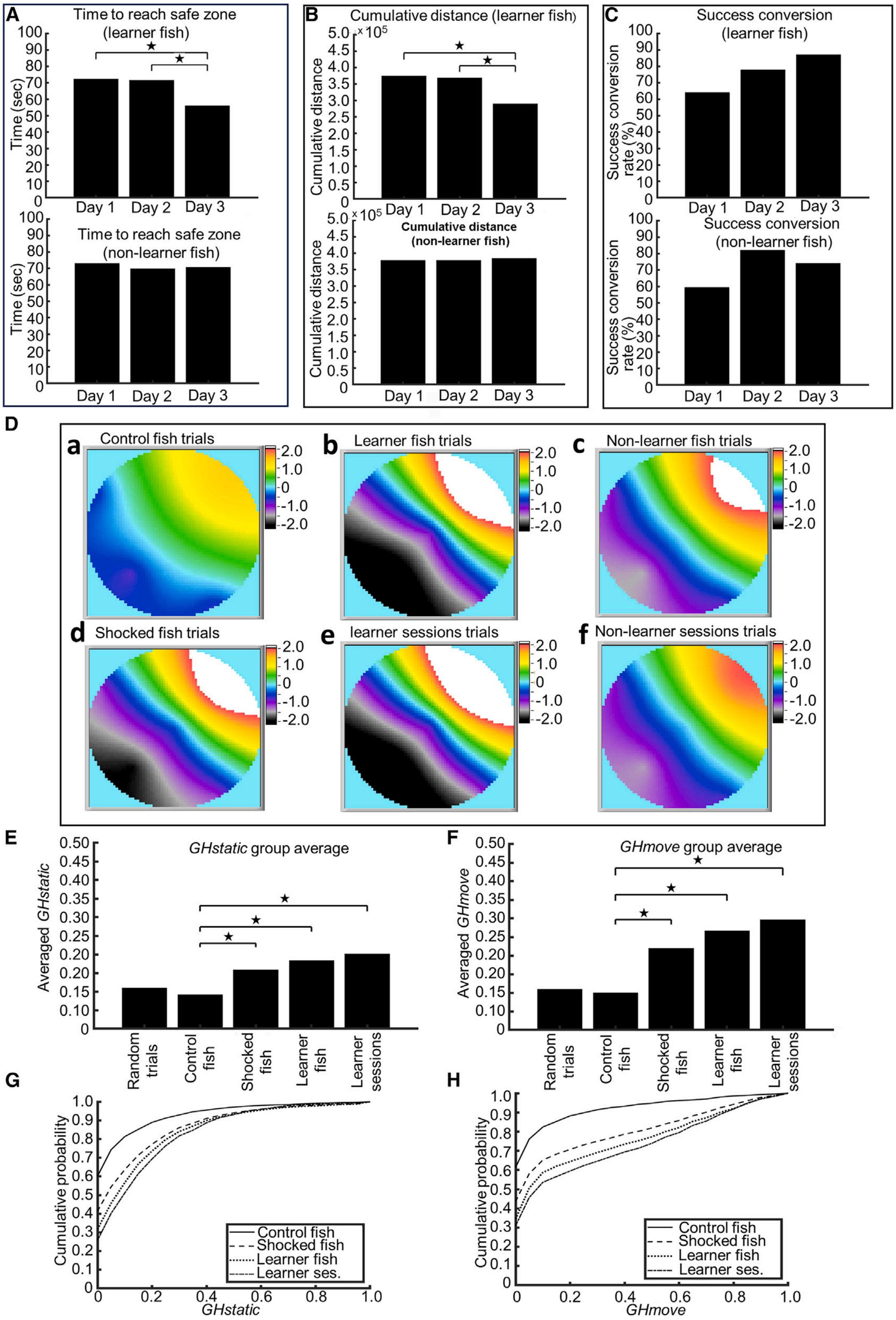

(legend on next page)

with only a few trials per day, head-fixed zebrafish in VR take many more trials to learn the location of the safe zone. Therefore, our focus was to check whether adult head-fixed zebrafish could achieve the spatial learning part of the MWM in VR, as that is the first requirement for future optical imaging. Nevertheless, we cannot rule out the presence of elements of associative learning in our VMWM protocol.

We assumed that to reach the safe zone in our VMWM experiment, zebrafish would use spatial learning based on distal cues that were placed outside the virtual tank, which requires similar brain function to what rodents use in the MWM. Furthermore, the condition for success in a VMWM trial required the zebrafish to be in the safe zone for five consecutive seconds rather than merely reaching the safe zone, which mimics the few seconds of time required in real-world MWM experiments for the experimenter to rescue the rodent that managed to find the platform. Finally, the size of the safe zone is no more than 6% of the virtual tank, which is comparable to the real-world Morris maze for rodents. These experimental settings make the VMWM an effective virtual version of the MWM, modified for zebrafish.

Results show that some zebrafish can learn the VMWM task by perceiving and remembering locations within the virtual space. The increased straightness in all starting positions over days and the decrease in time required for success and cumulative distance suggest that zebrafish got increasingly more used to finding the invisible safe zone over days. Besides, the results of persistence and *GH* analysis strongly indicate that the high success rate of fish in the shocked group was achieved by understanding the rule and learning the VMWM task, with higher motivation for escaping from receiving the periodic shock. The increase in motivation for reaching the safe zone was evident in the increased speed of the shocked group compared to the control group. Overall, we conclude that adult zebrafish could learn the VMWM task by manifesting spatial learning ability. We assume that future optical imaging studies with our VMWM task can reveal the neural mechanism of novel spatial learning, which may involve location-specific firing of cells in zebrafish brains. Furthermore, our VR system can be an essential platform for future experiments, including 2D navigation in complex environments, planning and decision-making, and social behavior of adult zebrafish.

Limitations of the study

This study presents a 2D VR system for head-fixed adult zebrafish. It is known that teleosts like zebrafish use their head as much as their tail for turning, and therefore head fixation can disrupt the physical mechanism of movement, though a gradual adaptation to the head-fixed state and the artificial movement mechanism is expected from the zebrafish. We do not presume that all zebrafish have such an ability, i.e., there seems to be a large individual difference. Based on our observations, some zebrafish may also show a "freezing" effect when an electric shock is provided. We think that more state-of-the-art display systems, like bending organic displays and providing somatosensory feedback using water flow or vibrators, could be added to make a more realistic VR system. Finally, due to the speed of our imaging system ($\sim$10 Hz), we did not implement a higher speed in the detection of tail movements and update of the VR. However, the existing system can be enhanced programmatically to implement higher speed of processing.

RESOURCE AVAILABILITY

Lead contact

Further information may be directed to and will be fulfilled by the lead contact, Hitoshi Okamoto (hitoshi.okamoto@riken.jp).

Materials availability

The study did not generate any unique reagents.

Data and code availability

- Data generated in this study are available from the lead author upon reasonable request and without restriction.
- The custom codes written in LabVIEW and MATLAB used in this research to create and control the VR system can be found in the repository (https://doi.org/10.5281/zenodo.10703218). The lead author can provide custom codes for the analysis of results upon request.
- Any additional information required to reproduce the data reported in this study is available from the lead contact upon request.

ACKNOWLEDGMENTS

This work was supported by MEXT KAKENHI Grant-in-Aid (H.O., 21H04814, 22H05520, and 23H04976) and partially supported by the RIKEN-Kao Collaboration Center.

AUTHOR CONTRIBUTIONS

Conceptualization, T.I. and H.O.; methodology, T.I. and M.T.; software, T.I.; formal analysis, T.I.; Investigation, T.I.; resources, M.T. and Y.T.; writing – original draft, T.I.; writing – review & editing, T.I. and H.O.; funding acquisition, H.O.

Figure 5. Characteristics of learning, persistence maps, and goal headedness in VMWM

(A) Top: average time for success (time taken to reach the safe zone) for success trials pooled from learner fish over days. Bottom: average time for success (time taken to reach the safe zone) for success trials pooled from non-learner fish over days.

(B) Top: average cumulative distance for success trials pooled from learner fish over days. Bottom: average cumulative distance for success trials pooled from non-learner fish over days.

(C) Top: success conversion rate by day from trials pooled from learner fish. Bottom: success conversion rate by day from trials pooled from non-learner fish.

(D) Persistence heatmaps derived from pooled trials of control fish (a), shocked fish (d), learner (b) and non-learner (c) fish, and learner (e) and non-learner (f) sessions in VMWM task. Red and white colors show high persistence.

(E) Average value of *GHstatic* of trials pooled from simulated random trials, control fish, shocked fish, learner fish, and learner sessions. *GHstatic* significantly increases between control fish and shocked fish groups.

(F) Average value of *GHmove* of trials pooled from simulated random trials, control fish, shocked fish, learner fish, and learner sessions. *GHmove* significantly increases between the control fish and shocked fish groups.

(G) Cumulative probability distribution of *GHstatic* values for control fish, shocked fish, learner fish, and learner sessions.

(H) Cumulative probability distribution of *GHmove* values for control fish, shocked fish, learner fish, and learner sessions.

DECLARATION OF INTERESTS

The authors declare no competing interests.

STAR★METHODS

Detailed methods are provided in the online version of this paper and include the following:

- KEY RESOURCES TABLE
- EXPERIMENTAL MODEL AND STUDY PARTICIPANT DETAILS
- METHOD DETAILS
  - Fixation of living adult zebrafish in virtual reality
  - Virtual reality with LabVIEW and unity
  - The Virtual Morris Water Maze-like (VMWM) task
  - Straightness index (SI)
  - Cumulative distance
  - Success conversion rate
  - Persistence toward goal
  - Goal headedness (GH)
- QUANTIFICATION AND STATISTICAL ANALYSIS

SUPPLEMENTAL INFORMATION

Supplemental information can be found online at https://doi.org/10.1016/j.crmeth.2024.100863.

Received: March 26, 2024
Revised: June 11, 2024
Accepted: August 30, 2024
Published: September 23, 2024

REFERENCES

1. Torigoe, M., Islam, T., Kakinuma, H., Fung, C.C.A., Isomura, T., Shimazaki, H., Aoki, T., Fukai, T., and Okamoto, H. (2021). Zebrafish capable of generating future state prediction error show improved active avoidance behavior in virtual reality. Nat. Commun. *12*, 5712. https://doi.org/10.1038/s41467-021-26010-7.
2. Holscher, C. (2005). Rats are able to navigate in virtual environments. J. Exp. Biol. *208*, 561–569. https://doi.org/10.1242/jeb.01371.
3. Harvey, C.D., Collman, F., Dombeck, D.A., and Tank, D.W. (2009). Intracellular dynamics of hippocampal place cells during virtual navigation. Nature *461*, 941–946. https://doi.org/10.1038/nature08499.
4. Minderer, M., Harvey, C.D., Donato, F., and Moser, E.I. (2016). Virtual reality explored. Nature *533*, 324–325. https://doi.org/10.1038/nature17899.
5. Reiser, M.B., and Dickinson, M.H. (2008). A modular display system for insect behavioral neuroscience. J. Neurosci. Methods *167*, 127–139. https://doi.org/10.1016/j.jneumeth.2007.07.019.
6. Kim, S.S., Rouault, H., Druckmann, S., and Jayaraman, V. (2017). Ring attractor dynamics in the Drosophila central brain. Science *356*, 849–853. https://doi.org/10.1126/science.aal4835.
7. Dombeck, D.A., and Reiser, M.B. (2012). Real neuroscience in virtual worlds. Curr. Opin. Neurobiol. *22*, 3–10. https://doi.org/10.1016/j.conb.2011.10.015.
8. Sato, M., Kawano, M., Mizuta, K., Islam, T., Lee, M.G., and Hayashi, Y. (2017). Hippocampus-dependent goal localization by head-fixed mice in virtual reality. eNeuro *4*, ENEURO.0369-16.2017. https://doi.org/10.1523/ENEURO.0369-16.2017.
9. Larsch, J., and Baier, H. (2018). Biological Motion as an Innate Perceptual Mechanism Driving Social Affiliation. Curr. Biol. *28*, 3523–3532.e4. https://doi.org/10.1016/j.cub.2018.09.014.
10. Stowers, J.R., Hofbauer, M., Bastien, R., Griessner, J., Higgins, P., Farooqui, S., Fischer, R.M., Nowikovsky, K., Haubensak, W., Couzin, I.D., et al. (2017). Virtual reality for freely moving animals. Nat. Methods *14*, 995–1002. https://doi.org/10.1038/nmeth.4399.
11. Dombeck, D.A., Khabbaz, A.N., Collman, F., Adelman, T.L., and Tank, D.W. (2007). Imaging Large-Scale Neural Activity with Cellular Resolution in Awake, Mobile Mice. Neuron *56*, 43–57. https://doi.org/10.1016/j.neuron.2007.08.003.
12. Maimon, G., Straw, A.D., and Dickinson, M.H. (2010). Active flight increases the gain of visual motion processing in Drosophila. Nat. Neurosci. *13*, 393–399. https://doi.org/10.1038/nn.2492.
13. Seelig, J.D., Chiappe, M.E., Lott, G.K., Dutta, A., Osborne, J.E., Reiser, M.B., and Jayaraman, V. (2010). Two-photon calcium imaging from head-fixed Drosophila during optomotor walking behavior. Nat. Methods *7*, 535–540. https://doi.org/10.1038/nmeth.1468.
14. Ahrens, M.B., Li, J.M., Orger, M.B., Robson, D.N., Schier, A.F., Engert, F., and Portugues, R. (2012). Brain-wide neuronal dynamics during motor adaptation in zebrafish. Nature *485*, 471–477. https://doi.org/10.1038/nature11057.
15. Picardo, M.A., Merel, J., Katlowitz, K.A., Vallentin, D., Okobi, D.E., Benezra, S.E., Clary, R.C., Pnevmatikakis, E.A., Paninski, L., and Long, M.A. (2016). Population-Level Representation of a Temporal Sequence Underlying Song Production in the Zebra Finch. Neuron *90*, 866–876. https://doi.org/10.1016/j.neuron.2016.02.016.
16. Dombeck, D.A., Harvey, C.D., Tian, L., Looger, L.L., and Tank, D.W. (2010). Functional imaging of hippocampal place cells at cellular resolution during virtual navigation. Nat. Neurosci. *13*, 1433–1440. https://doi.org/10.1038/nn.2648.
17. Royer, S., Zemelman, B.V., Losonczy, A., Kim, J., Chance, F., Magee, J.C., and Buzsáki, G. (2012). Control of timing, rate and bursts of hippocampal place cells by dendritic and somatic inhibition. Nat. Neurosci. *15*, 769–775. https://doi.org/10.1038/nn.3077.
18. Domnisoru, C., Kinkhabwala, A.A., and Tank, D.W. (2013). Membrane potential dynamics of grid cells. Nature *495*, 199–204. https://doi.org/10.1038/nature11973.
19. Schmidt-Hieber, C., and Häusser, M. (2013). Cellular mechanisms of spatial navigation in the medial entorhinal cortex. Nat. Neurosci. *16*, 325–331. https://doi.org/10.1038/nn.3340.
20. Low, R.J., Gu, Y., and Tank, D.W. (2014). Cellular resolution optical access to brain regions in fissures: imaging medial prefrontal cortex and grid cells in entorhinal cortex. Proc. Natl. Acad. Sci. USA *111*, 18739–18744. https://doi.org/10.1073/pnas.142175311.
21. Villette, V., Malvache, A., Tressard, T., Dupuy, N., and Cossart, R. (2015). Internally Recurring Hippocampal Sequences as a Population Template of Spatiotemporal Information. Neuron *88*, 357–366. https://doi.org/10.1016/j.neuron.2015.09.052.
22. Cohen, J.D., Bolstad, M., and Lee, A.K. (2017). Experience-dependent shaping of hippocampal CA1 intracellular activity in novel and familiar environments. Elife *6*, e23040. https://doi.org/10.7554/eLife.23040.
23. Heys, J.G., Rangarajan, K.V., and Dombeck, D.A. (2014). The functional micro-organization of grid cells revealed by cellular resolution imaging. Neuron *84*, 1079–1090. https://doi.org/10.1016/j.neuron.2014.10.048.
24. Chen, G., King, J.A., Burgess, N., and O'Keefe, J. (2013). How vision and movement combine in the hippocampal place code. Proc. Natl. Acad. Sci. USA *110*, 378–383. https://doi.org/10.1073/pnas.1215834110.
25. Acharya, L., Aghajan, Z.M., Vuong, C., Moore, J.J., and Mehta, M.R. (2016). Causal influence of visual cues on hippocampal directional Selectivity. Cell *164*, 197–207. https://doi.org/10.1016/j.cell.2015.12.015.
26. Aghajan, Z.M., Acharya, L., Moore, J.J., Cushman, J.D., Vuong, C., and Mehta, M.R. (2015). Impaired spatial selectivity and intact phase precession in two-dimensional virtual reality. Nat. Neurosci. *18*, 121–128. https://doi.org/10.1038/nn.3884.
27. Aronov, D., and Tank, D.W. (2014). Engagement of neural circuits underlying 2D spatial navigation in a rodent virtual reality system. Neuron *84*, 442–456. https://doi.org/10.1016/j.neuron.2014.08.042.

CellPress
OPEN ACCESS

28. Portugues, R., and Engert, F. (2011). Adaptive locomotor behavior in larval zebrafish. Front. Syst. Neurosci. *5*, 72. https://doi.org/10.3389/fnsys.2011.00072.

29. Ahrens, M.B., Orger, M.B., Robson, D.N., Li, J.M., and Keller, P.J. (2013). Whole-brain functional imaging at cellular resolution using light-sheet microscopy. Nat. Methods *10*, 413–420. https://doi.org/10.1038/nmeth.2434.

30. Portugues, R., Severi, K.E., Wyart, C., and Ahrens, M.B. (2013). Optogenetics in a transparent animal: circuit function in the larval zebrafish. Curr. Opin. Neurobiol. *23*, 119–126. https://doi.org/10.1016/j.conb.2012.11.001.

31. Friedrich, R.W., Jacobson, G.A., and Zhu, P. (2010). Circuit Neuroscience in Zebrafish. Curr. Biol. *20*, R371–R381. https://doi.org/10.1016/j.cub.2010.02.039.

32. Muto, A., Ohkura, M., Abe, G., Nakai, J., and Kawakami, K. (2013). Real-Time Visualization of Neuronal Activity during Perception. Curr. Biol. *23*, 307–311. https://doi.org/10.1016/j.cub.2012.12.040.

33. Kim, D.H., Kim, J., Marques, J.C., Grama, A., Hildebrand, D.G.C., Gu, W., Li, J.M., and Robson, D.N. (2017). Pan-neuronal calcium imaging with cellular resolution in freely swimming zebrafish. Nat. Methods *14*, 1107–1114. https://doi.org/10.1038/nmeth.4429.

34. Valente, A., Huang, K.-H., Portugues, R., and Engert, F. (2012). Ontogeny of classical and operant learning behaviors in zebrafish. Learn. Mem. *19*, 170–177. https://doi.org/10.1101/lm.025668.112.

35. Huang, K.-H., Rupprecht, P., Frank, T., Kawakami, K., Bouwmeester, T., and Friedrich, R.W. (2020). A virtual reality system to analyze neural activity and behavior in adult zebrafish. Nat. Methods *17*, 343–351. https://doi.org/10.1038/s41592-020-0759-2.

36. Morris, R.G., Garrud, P., Rawlins, J.N., and O'Keefe, J. (1982). Place navigation impaired in rats with hippocampal lesions. Nature *297*, 681–683. https://doi.org/10.1038/297681a0.

37. Quach, K.Q., Kim, H., Nguyen, T.V., Xia, C., Cho, W.S., and Chon, T.S. (2015). Inferring variance and invariance in multi-individual behavior of zebrafish (Danio rerio) responding to chemical stress. Anim. Cell Syst. *19*, 252–261. https://doi.org/10.1080/19768354.2015.1054308.

38. Chen, X., and Engert, F. (2014). Navigational strategies underlying phototaxis in larval zebrafish. Front. Syst. Neurosci. *8*, 39. https://doi.org/10.3389/fnsys.2014.00039.

39. Lee, J.G., and McShea, D.W. (2020). Operationalizing goal directedness: An empirical route to advancing a philosophical discussion. Philos. Theor. Pract. Biol. *12*, 005. https://doi.org/10.3998/ptpbio.16039257.0012.005.

40. Chow, D.M., Sinefeld, D., Kolkman, K.E., Ouzounov, D.G., Akbari, N., Tatarsky, R., Bass, A., Xu, C., and Fetcho, J.R. (2020). Deep three-photon imaging of the brain in intact adult zebrafish. Nat. Methods *17*, 605–608. https://doi.org/10.1038/s41592-020-0819-7.

41. Vorhees, C.V., and Williams, M.T. (2006). Morris water maze: procedures for assessing spatial and related forms of learning and memory. Nat. Protoc. *1*, 848–858. https://doi.org/10.1038/nprot.2006.116.

42. Chen, G., King, J.A., Lu, Y., Cacucci, F., and Burgess, N. (2018). Spatial cell firing during virtual navigation of open arenas by head-restrained mice. Elife *7*, e34789. https://doi.org/10.7554/eLife.34789.

STAR★METHODS

KEY RESOURCES TABLE

REAGENT or RESOURCE	SOURCE	IDENTIFIER
Software and algorithms		
LabVIEW	National Instruments	https://www.ni.com/en.html
MATLAB	MathWorks	https://www.mathworks.com/
Unity Engine	Unity	https://unity.com/
Visual Studio	Microsoft	https://visualstudio.microsoft.com/

EXPERIMENTAL MODEL AND STUDY PARTICIPANT DETAILS

All surgical and experimental procedures were reviewed and approved by the Animal Care and Use Committees of the RIKEN Center for Brain Science. Zebrafish (Danio rerio) were bred and raised under standard conditions. In this study, we used wild-type zebrafish (RIKEN Wako strain) aged >6 months from both sexes without bias.

METHOD DETAILS

Fixation of living adult zebrafish in virtual reality

A custom-made platform lying at the center of a round transparent acryl tank was used to fixate the adult zebrafish (Figure 1A). Zebrafish were briefly anesthetized with 0.02% tricaine (ethyl 3-aminobenzoate methane sulfonate salt, Sigma-Aldrich) diluted in fish rearing water and mounted on a hand-made surgery apparatus. During surgery, fish-rearing water with 0.02% tricaine was continuously perfused to keep the fish alive and maintain anesthesia. First, the skin above the skull over the telencephalon and the tectum was removed using micro knives (10315-12, Fine Science Tools; Figure 1A–1a). After drying the surface, a dental bond (Scotchbond Universal Adhesive, 41255, 3I) was pasted on the skull using a toothpick and illuminated with blue LED light for 10 s (Figure 1A-b). Next, A stainless rod of radius 0.2mm was placed on the skull (Figure 1A–c), and then fluidic dental cement (Filtek Supreme Ultra, 6032XW, 3I) was placed over the rod on the skull (Figure 1A–d). Blue LED light was used to harden the cement and fix the rod on the skull. The base of the fixation apparatus, the assembled harness, and the plastic ceiling to hold the fish body were set (Figure 1A–e). The tips of the rod were inserted into the slits in the small part of the assembled harness and fixed with screws to ensure that the rod did not rotate, and that the fish body remained horizontal. After recovery from the anesthesia by perfusion with fish-rearing water, the head-fixed fish on the fixation platform (Figure 1A–f) was transferred to the virtual reality environment. Two electrodes were placed on either side of the fish body to provide electrical shock. After the end of the experiment, zebrafish were released in another tank with the metallic rod still fixed on the head. If the fish was used for an experiment the next day, it was again brought to the VR setup, and the tips of the rod were fixed with the assembly. A head-fixated zebrafish on the white-colored fixation platform with the rod on the head is seen in Figure 1B.

Virtual reality with LabVIEW and unity

The head-fixed zebrafish situated on the fixation platform was placed inside a transparent circular tank filled with water (Figure 1C). The virtual reality environment consisted of four LCDs (Good display, Model No. GD70MLXD, native resolution 1280*1024, 7-inches screen size) placed on the left, front, right, and bottom of the fish tank. VR space was created using Unity 2017, a free version on a ‘VR display’ computer featuring an NVIDIA graphics board with four display outputs. A first-person view with four virtual cameras (Figures 2C and 4D) corresponding to the four displays mentioned above was used. A custom-made control program made with LabVIEW (National Instruments) and MATLAB (MathWorks) residing in a ‘control’ computer was used to record the tail movement and calculate forces for forward motion and turning. The force values were communicated to a Unity script residing in a ‘VR display’ computer, and information about the fish in virtual reality (position, head direction, collision, etc.) was communicated between the two computers using TCP/IP. The tail movement was captured by an HD USB camera (Hardware ID 9230, Vendor: ARC International). The fish’s body was illuminated with an infrared LED light (910 nm, intelligent LED solutions). A Thorlabs FBH940-10 bandpass filter was attached to the camera’s lens to exclude interference from the display light and pass light only from the illuminated fish body and surroundings. Because of the contrast between the fish body and the white colored platform beneath, the body of the fish could be detected with binary thresholding, using the camera placed at the top of the tank in such a manner that the body of the fish was along the vertical (Y) axis of the image taken by the camera. For each time frame of 100ms, a centerline lying along the body was calculated by connecting the midpoints of the body in every row of the image. The centerline’s upper points corresponded to the body’s upper portion. We took the three uppermost points of the centerline and an average of these three points was used to calculate the position of the upper body. Similarly, the lowermost three points of the straight line were averaged to find the position of the

tail end. The difference between the position of the tail end and the upper body was calculated along the X axis to see the amount of deviation of the tail from the midline. This deviation of the tail end with reference to the upper body (the deviation had positive and negative values corresponding to right and left side deviation) was calculated in each time step. Furthermore, we observed that there was bias in the deviation depending on the fish; that is, the resting position of the tail end was biased to either right or left of the upper body for many fish over a certain period during the experiment. The direction of bias changed over time. We, therefore, took a comparatively large moving time window of 10s (1000 data points) and averaged the deviation of the tail end in that window to set a 'reference mid-position' of the tail. For each time step, the difference of the tail end with respect to the the-then 'reference mid-position' was calculated to find the amount of movement of the tail. Because there was noise in the image taken by the camera due to fish movement and minor change of focus due to light condition and little vertical movement of the tail, a threshold value (usually 5–7 pixels) was used to define if the movement was real, that is, this threshold was subtracted from the movement amount to find the 'actual movement' of the tail. This refined tail movement caused an oscillation when the fish moved its tail spontaneously. A Fourier transformation (FFT) of this oscillation was used to calculate the high frequency ration (HFR), basically the proportion of higher frequency amplitudes of FFT in all summed frequency amplitudes. In our system, the system was operated at 10Hz to keep synchrony with the two-photon microscope for future use. Therefore, the active detectable frequency of tail oscillation varied between 0 and 5Hz, and for calculation of HFR, we took the proportion of FFT amplitudes ranging between 1 and 5 Hz. Notably, the frequency range for HFR calculation can be modified if the system's operating frequency is higher. With the HFR value, subsequent force values for forward movement ($Force_{translation}$) and turning ($Force_{rotation}$) were calculated with the following Equations 2 and 3:

$$Force_{translation} = gain_{translation} \times \{(HFR(t) - HFR_{min}) \times 10\}^2 \quad \text{(Equation 2)}$$

$$Force_{rotation} = gain_{rotation} \times \{(HFR_{max} - HFR(t)) \times 10\}^2 \quad \text{(Equation 3)}$$

Here, $gain_{translation}$ is the gain value for speed, which was set within the range of 0.5–1, while $gain_{rotation}$ is the gain value for turn which was set from the range of 0.1–0.15. These gain values were set during the initial movement of fish in the VR, considering the agility and body size. Values of HFR_{min} and HFR_{max} correspond to the minimum and maximum values of HFR. For each fish, the values of HFR_{min} and HFR_{max} were updated continuously throughout the experiment. Typical values of HFR_{min} was near zero, HFR_{max} was between 0.7–0.8. The use of nonlinearity and mutual exclusiveness of the two force values are shown in Figure 1D.

The direction of turn was determined in the following manner. If the two successive last positions of the end of the tail was on the right of the 'reference mid-position', then the turning direction was rightwards (clockwise). The same criteria were used for leftwards (counterclockwise) turns. The two force values for forward movement and turning, as well as the turn direction and other variables, were calculated within a custom control program written in LabVIEW and MATLAB in the 'control' computer, which communicated the values to a Unity script (in 'VR display' computer) through TCP/IP, using Unity engine. Inside Unity, the force values were applied to a point mass with attached virtual cameras, using the physics properties (Mass = 1, drag = 1, Angular drag = 10000), which enabled an automatic inertia effect during the start and the end of a movement. The rigid body setup, along with use of the physics engine, compelled the zebrafish to change directions in VR with tail movements whenever they collided with the boundary wall inside the VR. A National Instruments USB DAQ (NI-6002) was used to provide electric shock to the fish through electrodes placed on both sides of the vicinity of the head of the fish. The two electrodes did not touch the fish body. The DAQ device had an output voltage range of 0–5 V, and it was used to generate 0.5–1 V for electric shock to the zebrafish. The resultant current flew between the two electrodes through water and the fish body. The amount of current depended on the purity of the water and the size of the fish, but it was enough to be an aversive stimulus causing visible discomfort to the zebrafish. With these, zebrafish could experience forward movement and turning through virtual space with a first-person view.

The Virtual Morris Water Maze-like (VMWM) task

In the VMWM task, a 4-virtual-camera system was used for the first-person view. To provide the first-person view, a point-object (called an empty object in Unity) contained four virtual cameras for left, front, right, and bottom view (Figure 2D). Force values communicated from the control program were applied to the empty object to make it have a forward speed and turning speed, along with the cameras. The front camera had a horizontal view of 90° (plus or minus 45° on left and right). The left and right cameras also each had a 90-degree view; thus, a total of 270 degrees of field of view was provided to the zebrafish. The vertical position of the empty object containing the cameras was at 50 units. The bottom camera provided a view of the floor from the height of the fish. The output of the four virtual cameras was shown on the four displays simultaneously. The arena for VMWM had a radius of 335 units. The boundary wall had the height of 100 units, with a uniform black texture (Figures 2A–2D). The boundary wall was set as impenetrable and therefore zebrafish had to turn away from the wall whenever they collided with it. The floor had a chessboard texture to ensure the zebrafish understood its movement in virtual space. Six pillar-shaped objects with different colors (Figures 2A and 2B) were placed outside the area to act as distal cues. A brown textured cylinder-shaped platform (safe zone) with a radius of 80 units was placed so that its center corresponded to the location X = 160, Z = 160. The platform was made invisible from the beginning of a trial and was made visible only when the fish came onto it, to provide the fish with a confirming visual cue instead of the somatosensory cue the rodents receive in the real-world Morris Water Maze when they reach the platform. However, if the fish moved out of the safe zone, it became invisible again. One trial lasted for 2 min, with a 30-s inter-trial interval. Movement in the VR was not allowed during the interval time regardless of tail

movements. The initial position at the beginning of a trial varied among four positions (Figure 2C, Position 1= (160, −292), Position 2= (−160, −160), Position 3= (−292,160) and Position 4= (0,0)) and the initial head direction was randomly chosen in the range of 0∼360°. Among these, Positions 1–3 were equidistant from the center of the safe zone and were in three different quadrants that did not include the quadrant of the safe zone. Position 4 was introduced to some of the zebrafish of the shocked group for comparison of success performance with the other three positions. To mimic the presence of water as an aversive condition in the original Morris Water Maze, we used a periodic electric shock of 0.5–1 V. The zebrafish of the shocked group received this periodic shock for 300 ms every 2 s from the start of the trial until they reached the safe zone. Electric shock ceased as soon as the zebrafish entered the safe zone. Cessation of the periodic shock by reaching the safe zone was the reward for zebrafish in the shocked group. However, the zebrafish of the control group did not receive the periodic electric shock and therefore received no reward. If the zebrafish stayed in the safe zone for five consecutive seconds, the trial was considered a success, and further movement in the rest of the time of that trial was not allowed. The length of 5 s was introduced to mimic the time length of few seconds that is required by the experimenter to manually recover the rodent from the safe platform in real-world Morris Water Maze. The artificial 'stalling' of movement after 5 s was used to consolidate the understanding of the learning rule in zebrafish. No movement in virtual space was allowed in the interval period, and no shock was given. Usually, a break of 10 min was provided after ten consecutive trials. On each day, roughly 50–80 trials were conducted, barring days when malfunctions like zebrafish escaping from fixation accidentally occurred. Before the start of the experimental VMWM trials, 1 h of free moving in the same VMWM arena (without the safe zone present) was performed by both control and shocked fish. This free-moving session aimed to enable the zebrafish to get used to VR. A VMWM trial was considered a success if the fish either stayed in the safe zone for five consecutive seconds with further movement stalling or the last position of the fish was in the safe zone. At the end of the experiment of a day, zebrafish were released from fixation with the metallic rod fixed to the head and were kept in another tank overnight.

Straightness index (SI)

To find whether the zebrafish could adapt to the two-dimensional VR mechanism and generate straighter paths in success trials over time, we defined an index called Straightness Index (SI). For a trajectory of the fish, if the Euclidean distance between the first and the last position of the fish is D, and the total distance covered by the trajectory is L, then the following Equation 4 defines the straightness index ranging from 0 to 1:

$$SI = \frac{D}{L} \quad \text{(Equation 4)}$$

As we can see from the above equation, SI will have higher values for paths that are straighter. The SI is used to measure the path straightness in success trials of the VMWM experiment.

Cumulative distance

The measure of cumulative distance was conducted in success trials by adding up the distance between the position of the zebrafish in the virtual arena and the center of the safe zone. In each trial, distance values were measured every time frame until the fish met the condition of success. A summation of these distance values for the whole trial length was considered as the cumulative distance of that trial.

Success conversion rate

To check if the understanding of the VMWM rule for success is learned by zebrafish, we calculated the 'success conversion rate' which is a ratio of the number of success trials and the number of trials in which the fish made at least one entry into the safe zone, therefore implying how the zebrafish has learnt to convert an entry to the safe zone into a success.

Persistence toward goal

We introduced a measure of persistence to check if the shocked zebrafish made a more extensive effort to reach the safe zone than the control fish in the VMWM task. First, we defined 3405 point-places inside the VMWM arena by using a grid of points. For a time-length of 0-T (T = trial length of VMWM), persistence toward a point-place A in a VMWM trial can be defined by a mean distance reduction toward that place with the following equation:

$$P(A) = \frac{\sum_{t=0}^{T} \left(D_{A(t-1)} - D_{A(t)}\right)}{Nmov} \quad \text{(Equation 5)}$$

Here, $D_{A(t-1)}$ is the distance from the position at time *(t-1)* to place A, $D_{A(t)}$ is the distance from the position at time *(t)* to place A, and $Nmov$ is the number of frames when $\left|D_{A(t-1)} - D_{A(t)}\right| \neq 0$, to not include to time points when distant has not changed from the previous frame.

After calculating persistence toward all the point places, a persistence map of the trial is found. Persistence defined above can have a positive or negative value, with a positive value showing some amount of persistence. Persistence maps of individual trials from a group of fish were averaged to find the average persistence maps (Figure 5D). For example, to see the persistence map of

control fish, all control fish trials were gathered, and persistence maps of individual trials were calculated. The average of these maps is shown as the control fish persistence map. Persistence maps for shocked, learner fish, and learner sessions were generated similarly. We found that a combination of the four start positions used in the VMWM experiment, and a trial length of 2 min caused a bias toward the upper right quadrant of the circular arena in the persistence map (Figure S1A, left most map of random trials) of 1200 artificially simulated trials. These simulated trials were created by constructing artificial trajectories with randomly picked speed and turning values from histograms of Zebrafish data. The random trial maps for each of the four positions (300 random trials for each position) are shown in Figure S1A lower row, and their summation forms the map for random trials in Figure S1A. However, the bias toward the upper right quadrant gradually disappeared with increasing trial length (Figure S1A, upper row).

Goal headedness (GH)

We defined the measure of goal-headedness (GH) to check how much zebrafish movement was directed toward the safe zone. This concept is illustrated in Figure S1C. In a single VMWM trial, the zebrafish can have multiple positions and head directions. If the extended line of the head direction, as shown in Figure S1C, goes through the safe zone (in the case of position P4), it is counted as a goal-directed orientation. Next, we divided the positions into static positions (positions that did not change from the previous frame) and moving positions (positions with a change from the previous frame). We calculated *GHstatic* by dividing the number of goal-directed static positions by the number of total static positions. For moving frames, we calculated *GHmove* by dividing the number of goal-directed moving positions by the number of total moving positions. In our view, while higher *GHstatic* may emphasize attention or planning toward reaching the goal, higher *GHmove* may correspond to direct execution of goal-directed behavior. The *GHstatic* and *GHmove* values of each trial were calculated, and the average of these values for a fish group, for example, a control fish group, was calculated by taking the average of all trials from fish of that group. Previously described 1200 simulated VMWM trials were used to find the chance level values of *GHstatic* and *GHmove*. and is shown as ‘random trials’ in Figures 5E–5H, along with the average values of the control, shocked, learner fish, and learner session groups.

QUANTIFICATION AND STATISTICAL ANALYSIS

The following method was used to determine whether a session of VMWM shocked fish showed a significantly higher success rate than control fish. First, we picked the control fish session with the highest success rate on each day (Table S2). This resulted in control fish #01 on day 1 and day 2 (13.33% and 15%) and control fish#04 on day 3 (21.67%) being the reference sessions for day1–3 of training. To find significantly higher learning performance (success rate) for any shocked fish session of day1, that session was compared to the day 1 session of control fish #01. Same process was followed for any shocked fish session of day 2 (compared with control fish#01, day 2) and day 3 (compared with control fish#04, day 3).The method of comparison had three parts: (1) A binomial test to check if the shocked fish session had a significantly higher success rate than the reference control session of the same day, (2) A Kolmogorov-Smirnov test using MATLAB *kstest2()* function to check if the probability distribution of last ten trials success rate values (0, 0.1,0.2, ….0.9,1) of the shocked fish session has significantly higher values (having a smaller tail in cumulative probability distribution function) than the distribution of the reference session, and (3) Students’ t-test (as success rates have a normal distribution in VMWM experiment) using MATLAB function *ttest2()* to check if the last ten trials’ success rate values of the shocked fish session have significantly higher mean compared to the reference session. In all three tests, the p-value threshold was 0.0001, considering the conservative Bonferroni correction for ∼500 statistical tests. A shocked fish session was considered a significantly high success (denoted as ‘learner session’ in this paper) if statistical significance was observed in all three tests mentioned above. A shocked fish was considered a ‘learner fish’ in the paper if the last session of the fish was a learner session.

Cell Reports Methods, Volume *4*

Supplemental information

Adult zebrafish can learn Morris water maze-like tasks in a two-dimensional virtual reality system

Tanvir Islam, Makio Torigoe, Yuki Tanimoto, and Hitoshi Okamoto

Supplemental Information

Supplementary Figures

Figure S1. Persistence and goal headedness in the VMWM task.

Figure S2. Heatmaps of statistical P-values of comparison of persistence at point-places in the VMWM arena.

Supplementary Tables

Table S1. Experimental information and learning performances of the shocked fish in the VMWM.

Table S2. Experimental information and learning performances of the control fish in the VMWM.

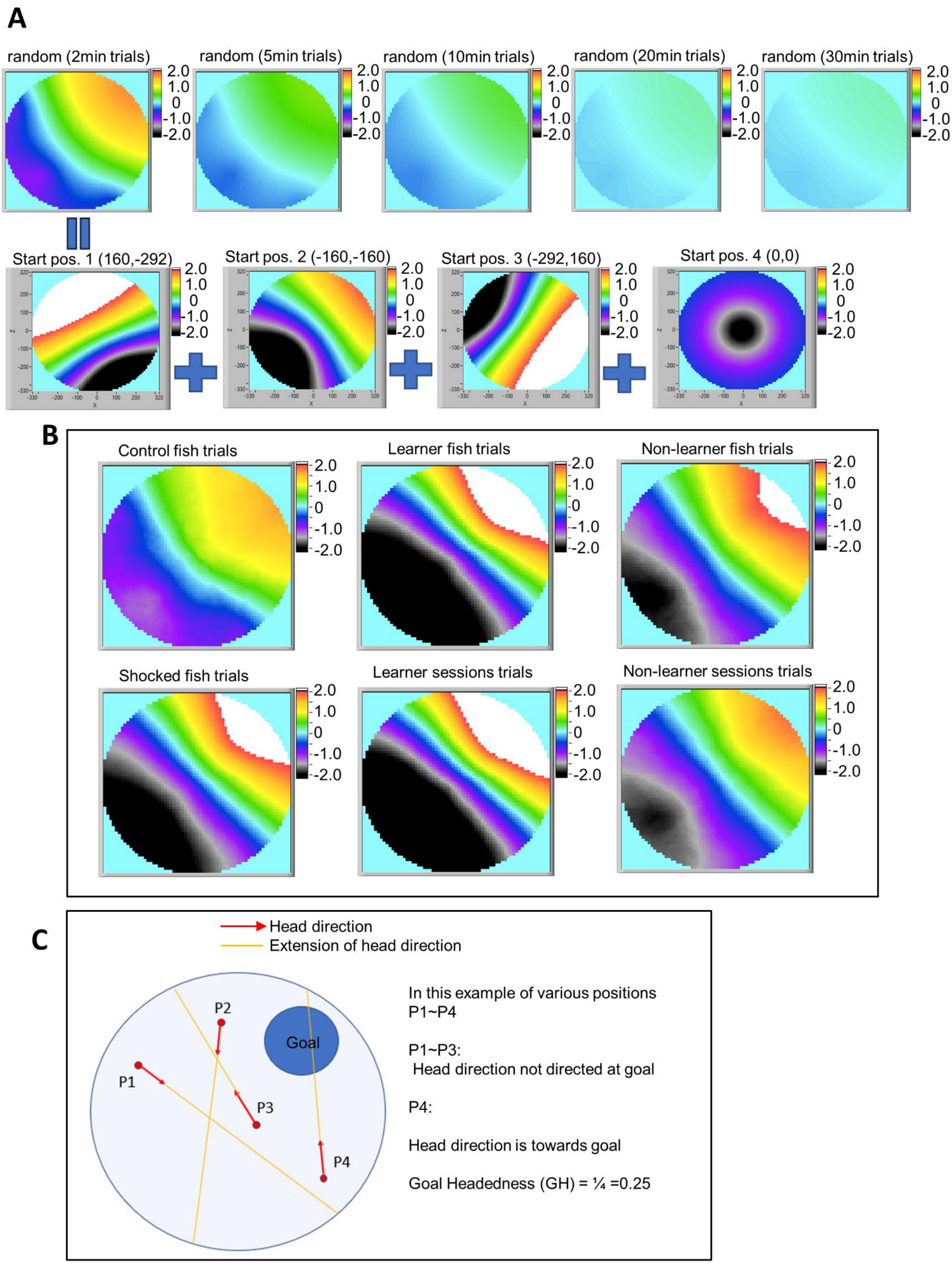
A
random (2min trials)
random (5min trials)
random (10min trials)
random (20min trials)
random (30min trials)
Start pos. 1 (160,-292)
Start pos. 2 (-160,-160)
Start pos. 3 (-292,160)
Start pos. 4 (0,0)
B
Control fish trials
Learner fish trials
Non-learner fish trials
Shocked fish trials
Learner sessions trials
Non-learner sessions trials
C
Head direction
Extension of head direction
P1
P2
P3
P4
Goal
In this example of various positions P1~P4
P1~P3:
Head direction not directed at goal
P4:
Head direction is towards goal
Goal Headedness (GH) = ¼ =0.25

Figure S1: Persistence and goal headedness in the VMWM task, related to Figure 5. (A) Persistence maps derived from 1200 simulated VMWM trials, using random movement. The first map from the upper left is for 2-minute trials, which have the same length as trials experienced by fish in VMWM trials. Because of the location of the start positions and the structure of the arena, as shown in the bottom four panels, there is a resultant bias of higher persistence towards the upper right quadrant of the arena. However, as the next four maps show, this bias disappears with increased length of trials. The calculation method of persistence is provided in the equation inside the text box. (B) Persistence heat maps derived by another method [40]. It is notable that the persistence maps look similar in two methods. (C) Explanatory diagram for calculation of goal-headedness (STAR Methods). Among the four positions p1-p4, the head direction during only position p4 is goal-headed, as the hypothetical extension if that head direction goes through the safe zone. In this case of four positions, the goal headedness score is therefore 1/4=0.25.

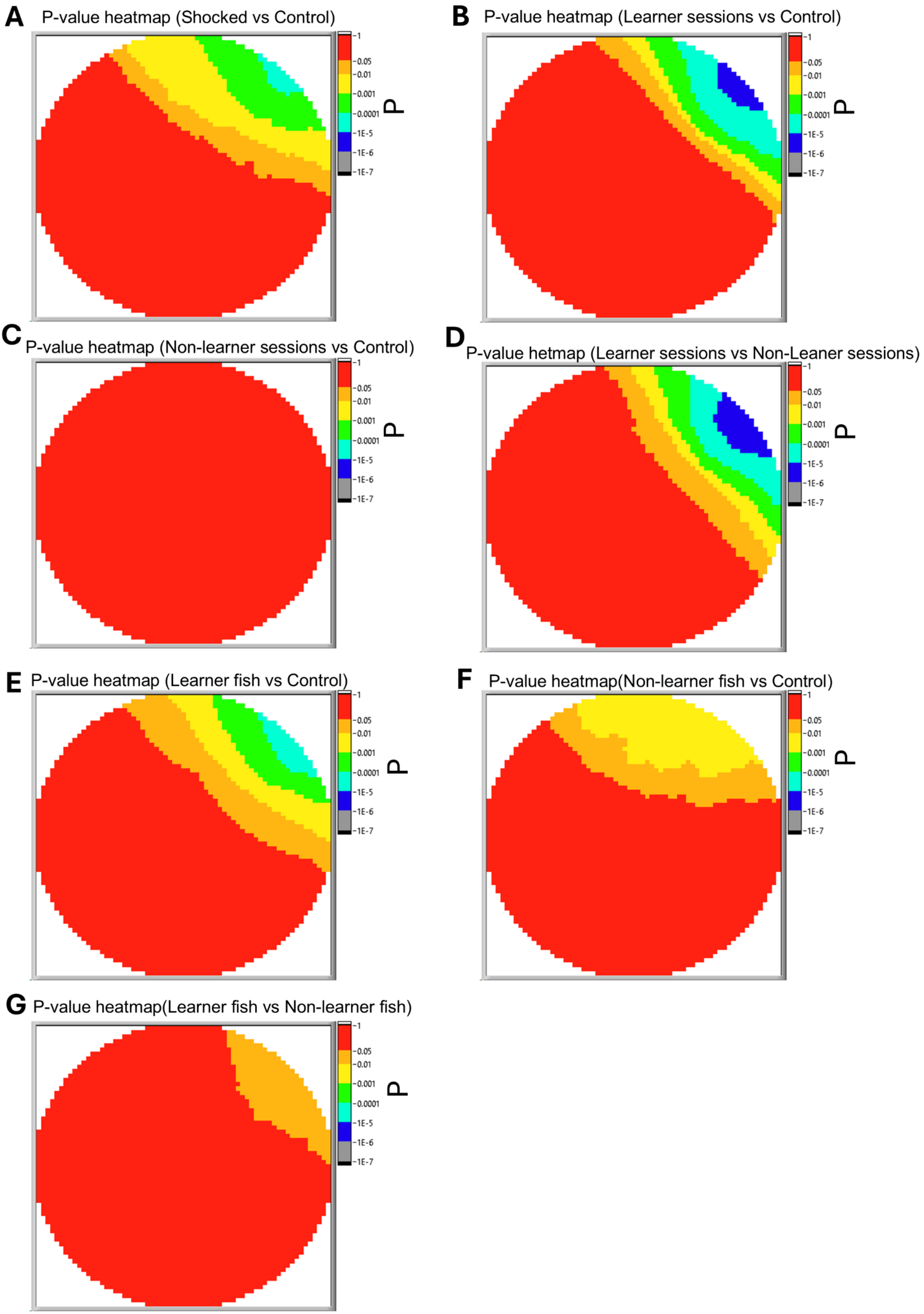
A
P-value heatmap (Shocked vs Control)
B
P-value heatmap (Learner sessions vs Control)
C
P-value heatmap (Non-learner sessions vs Control)
D
P-value hetmap (Learner sessions vs Non-Leaner sessions)
E
P-value heatmap (Learner fish vs Control)
F
P-value heatmap(Non-learner fish vs Control)
G
P-value heatmap(Learner fish vs Non-learner fish)
1
0.05
0.01
0.001
0.0001
1E-5
1E-6
1E-7
P

Figure S2: Heatmaps of statistical P-values at the point-places while comparing persistence from different groups, related to Figure 5. (A) P-value heatmap obtained from comparison of persistence in all point-places among the shocked and control fish (lower P-values mean higher significance). Shocked fish showed significantly higher persistence ($p<0.0001$) in the vicinity of the safe zone. (B) Similar P-value heatmap from comparison between learner sessions and control. Rest of the figures also show P-value heatmaps for the following statistical comparisons: (C) Non-learner sessions vs Control. (D) Learner sessions vs Non-learner sessions. (E) Learner fish vs Control. (F) Non-learner fish vs Control. (G) Learner fish vs Non-learner fish

Table S1: Experimental information and learning performances of the shocked fish in the VMWM, related to Figures 2 and 3. The number of trials, number of successes, and success rate are shown for each fish in the shocked group. In each cell corresponding to a fish and a day, a division of the number of success trials by the total number of trials is multiplied by 100 to show the success rate of that fish on that day. A (S) tag (S=Significant) is used if the success rate is significantly higher than control fish, otherwise a notation of (NS) is used. The sessions (day) with a label (S) are considered as “learner sessions” throughout the paper. A shocked fish is assigned an “L” or “learner” tag if the last session(day) of that fish is a “significant” session. Fish with an “L” tag are considered as “learner fish” throughout the paper.

Fish #	Day 1 success rate (SR) %	Day 2 success rate (SR) %	Day 3 success rate (SR)%	Day 4 success rate (SR) %	Learner? Learner=L
01	SR=4/70=5.71 P=0.2198 (NS)	SR=12/80=15 P=0.060456 (NS)	SR=6/65=9.23 P=0.35355 (NS)	N/A	
02	SR=16/70=22.85 P=0.020276 (NS)	SR=30/66=45.45 2.06E-08(S)	SR=0/60=0 P=1.48E-06 (NS)	N/A	
03	SR=19/65=29.23 P=0.000613(NS)	SR=31/65=47.69 P=4.78E-10 (S)	N/A	N/A	L
04	SR=44/70=62.8 2.12E-28(S)	N/A	N/A	N/A	L
05	SR=23/50=46 P=2.04E-08 (S)	SR=35/75=46.66 P=7.98E-11 (S)	N/A	N/A	L
06	SR=24/60=40 P=2.66E-07(S)	SR=32/60=53.33 P=5.55E-12(S)	N/A	N/A	L
07	SR=19/60=31.66 P=0.000197 (NS)	SR=7/60=11.66 P=0.3047(NS)	SR=2/60=3.33 P=8.36E-05(NS)	N/A	
08	SR=9/60=15 P=0.40721 (NS)	SR=28/60=46.66 P=6.03E-09(S)	SR=1/60=1.66 P=9.81E-06(NS)	N/A	
09	SR=19/70=27.14 P=0.001634 (NS)	SR=15/46=32.6 P=0.002164 (NS)	SR=30/51=58.82 P=1.23E-08(S)	SR=27/51=52.94 P=7.04E-07(S)	L
10	SR=29/54=53.7 P=2.26E-12 (S)	SR=17/65=26.15 P=0.013616 (NS)	N/A	N/A	
11	SR=23/73=31.5 p=4.83E-05(S)	SR=24/55=43.64 P=3.46E-07 (S)	SR=16/29=55.17 P=6.82E-05 (S)	N/A	L
12	SR=30/50=60 P=1.68E-14 (S)	SR=28/50=56 P=2.44E-11 (S)	SR=37/53=69.81 5.21E-14(S)	N/A	L
13	SR=30/80=37.5 P=5.13E-08 (S)	N/A	N/A	N/A	L
14	SR=17/80=21.25 P=0.03328 (NS)	N/A	N/A	N/A	
15	SR=34/80=42.5 P=1.35E-10 (S)	N/A	N/A	N/A	L
16	SR=48/80=60 P=1.90E-40 (S)	N/A	N/A	N/A	L
17	SR=15/80=18.75 P=0.10694 (NS)	SR=24/70=34.28 P=4.90E-05 (S)	N/A	N/A	L
18	SR=21/80=26.25 P=0.001519 (NS)	N/A	N/A	N/A	
19	SR=26/80=32.5 P=8.58E-06 (S)	N/A	N/A	N/A	L
20	SR=16/80=20 P=0.061609 (NS)	N/A	N/A	N/A	
21	SR=24/65=36.36 P=1.49E-06 (S)	SR=28/60=46.67 P=6.03E-09 (S)	SR=34/60=56.67 P=2.55E-09 (S)	N/A	L
22	SR=17/60=28.33 P=0.001695 (NS)	SR=32/60=49.23 P=8.84E-11 (S)	SR=25/60=41.66 P=0.0003 (NS)	N/A	
23	SR=5/80=6.25 P=0.035693 (NS)	SR=1/60=1.66 P=0.000675 (NS)	N/A	N/A	
24	SR=19/70=27.14 P=0.001634 (NS)	N/A	N/A	N/A	
25	SR=15/62=24.19% P=0.014621 (NS)	SR=4/64=6.25 P=0.027971(NS)	SR=45/86=52.32 9.39E-11(S)	N/A	L
26	SR=20/70=28.57 P=0.000617 (NS)	SR=29/60=48.33 P=1.16E-09 (S)	SR=20/60=33.33 P=0.020933 (NS)	N/A	
27	SR=6/70=8.57 P=0.15905 (NS)	N/A	N/A	N/A	

Table S2. Experimental information and learning performances of the control fish in the VMWM, related to Figures 2 and 3. Number of trials, number of successes, and success rate are shown for each fish in the control group. For any day, the control fish data with highest success rate (fish#01 for day 1 and day 2, fish#04 for day 3) is chosen to compare with any shocked fish data for statistical significance.

Fish #	Day 1 success rate (%)	Day 2 success rate (%)	Day 3 success rate (%)
01	8/60=13.33	9/60=15	3/60=5
02	6/60=10	8/60=13.33	2/60=3.33
03	6/60=10	1/60=1.66	10/60=16.67
04	2/60=3.33	4/60=6.66	13/60=21.67
05	0/80=0	6/70=8.57	N/A
06	2/80=2.5	6/70=8.57	5/70=7.14
07	2/70=2.85	7/70=10	1/70=1.42
08	6/70=8.57	5/70=7.14	4/70=5.71
09	4/70=5.71	4/70=5.71	1/70=1.42
10	5/70=7.14	1/70=1.42	0/70=0
